# Overexpression of tissue-nonspecific alkaline phosphatase (TNAP) in endothelial cells accelerates coronary artery disease in a mouse model of familial hypercholesterolemia

**DOI:** 10.1371/journal.pone.0186426

**Published:** 2017-10-12

**Authors:** Filippo Romanelli, AnthonyMarco Corbo, Maryam Salehi, Manisha C. Yadav, Soha Salman, David Petrosian, Omid J. Rashidbaigi, Jesse Chait, Jes Kuruvilla, Maria Plummer, Ilian Radichev, Kenneth B. Margulies, A. Martin Gerdes, Anthony B. Pinkerton, José Luis Millán, Alexei Y. Savinov, Olga V. Savinova

**Affiliations:** 1 Department of Biomedical Sciences, New York Institute of Technology College of Osteopathic Medicine, Old Westbury, New York, United States of America; 2 Children’s Health Research Center, Sanford Research, Sioux Falls, South Dakota, United States of America; 3 Human Genetics Program, Sanford Burnham Prebys Medical Discovery Institute, La Jolla, California, United States of America; 4 Department of Clinical Specialties, New York Institute of Technology College of Osteopathic Medicine, Old Westbury, New York, United States of America; 5 Heart Failure and Transplant Program, Perelman School of Medicine, University of Pennsylvania Translational Research Center, Philadelphia, Pennsylvania, United States of America; 6 Prebys Center for Drug Discovery, Sanford Burnham Prebys Medical Discovery Institute, La Jolla, California, United States of America; Brigham and Women's Hospital, Harvard Medical School, UNITED STATES

## Abstract

**Objective:**

Overexpression of tissue-nonspecific alkaline phosphatase (TNAP) in endothelium leads to arterial calcification in mice. The purpose of this study was to examine the effect of elevated endothelial TNAP on coronary atherosclerosis. In addition, we aimed to examine endogenous TNAP activity in human myocardium.

**Approach and results:**

A vascular pattern of TNAP activity was observed in human non-failing, ischemic, and idiopathic dilated hearts (5 per group); no differences were noted between groups in this study. Endothelial overexpression of TNAP was achieved in mice harboring a homozygous recessive mutation in the low density lipoprotein receptor (*whc* allele) utilizing a *Tie2-cre* recombinase (WHC-eTNAP mice). WHC-eTNAP developed significant coronary artery calcification at baseline compared WHC controls (4312 vs 0μm^2^ alizarin red area, p<0.001). Eight weeks after induction of atherosclerosis, lipid deposition in the coronary arteries of WHC-eTNAP was increased compared to WHC controls (121633 vs 9330μm^2^ oil red O area, p<0.05). Coronary lesions in WHC-eTNAP mice exhibited intimal thickening, calcifications, foam cells, and necrotic cores. This was accompanied by the reduction in body weight and left ventricular ejection fraction (19.5 vs. 23.6g, p<0.01; 35% vs. 47%, p<0.05). In a placebo-controlled experiment under atherogenic conditions, pharmacological inhibition of TNAP in WHC-eTNAP mice by a specific inhibitor SBI-425 (30mg*kg^-1^*d^-1^, for 5 weeks) reduced coronary calcium (78838 vs.144622μm^2^) and lipids (30754 vs. 77317μm^2^); improved body weight (22.4 vs.18.8g) and ejection fraction (59 vs. 47%). The effects of SBI-425 were significant in the direct comparisons with placebo but disappeared after TNAP-negative placebo-treated group was included in the models as healthy controls.

**Conclusions:**

Endogenous TNAP activity is present in human cardiac tissues. TNAP overexpression in vascular endothelium in mice leads to an unusual course of coronary atherosclerosis, in which calcification precedes lipid deposition. The prevalence and significance of this mechanism in human atherosclerosis requires further investigations.

## Introduction

Coronary artery calcification is an independent predictor of cardiovascular morbidity and mortality [1–8]. Despite our understanding of the clinical risks associated with cardiovascular calcification, the biological mechanism of interaction between vascular calcification and atherosclerosis (a major cause of cardiovascular disease) are poorly understood. Consequently, limited options are available to therapeutically target vascular calcification [9].

Tissue-nonspecific alkaline phosphatase (TNAP) promotes vascular calcification by regulating the levels of pyrophosphate (PP_i_), an inhibitor of mineralization. Upregulation of TNAP expression in vascular smooth muscle cells or in endothelial cells leads to pathological calcification in animal models [10, 11]. Increased TNAP expression has also been detected in patients suffering from vascular calcification associated with diabetes [12] and chronic kidney disease [13, 14]. Recent epidemiological studies point to a potential pathophysiological role and a prognostic value of increased blood alkaline phosphatase activity in human coronary artery disease [15, 16].

Circulating osteogenic endothelial progenitor cells measured in blood can serve as a biomarker for the severity of coronary artery disease [17–19]. It has also been established that endogenous endothelial TNAP activity is present in the arteries and arterial capillary borders in multiple species including humans, however the function of this enzyme in the endothelium is not completely understood. To explore the effects of osteogenic endothelial cells on the vasculature, we have recently developed a mouse model of pan-endothelial TNAP overexpression, in which were observed arterial calcification that was most prominent in the medium-sized arteries [11]. In this study, we hypothesized that under the conditions of hypercholesterolemia, vascular calcification within our transgenic endothelial TNAP model will increase lipid deposition into calcified coronary arteries. We also set out to evaluate a potential therapeutic approach for the reduction of coronary atherosclerosis through the use of a specific pharmacological TNAP inhibitor, SBI-425 [10, 20]. In addition we wished to confirm that endogenous TNAP activity is indeed present in human vasculature.

## Materials and methods

### Ethics statement

The human tissue samples were obtained from the Heart Failure and Transplant Program, Perelman School of Medicine of the University of Pennsylvania. All procedures were approved by the University of Pennsylvania institutional review board. Prospective informed consent for research use of human heart tissue were obtained from the appropriate next of kin of the donor by Gift of Life personnel. The human tissue research inquiry was received or recorded by Dr. Margulies; no protected health information that could be used to specifically identify the heart donor were available to other investigators.

Animal studies were approved by the Institutional Animal Care and Use Committees (IACUC) of Sanford Research (Sioux Falls, SD) and the New York Institute of Technology College of Osteopathic Medicine (Old Westbury, NY) and complied with the National Institutes of Health guidelines for humane treatment of laboratory animals. Mice were euthanized by exsanguination under 5% isoflurane in oxygen, *via* inhalation, or by carbon dioxide inhalation.

### Human myocardial samples

Human myocardium tissues were obtained from patients undergoing cardiac transplantation (diagnosed with ischemic and idiopathic dilated heart failure) or from donor hearts deemed unsuitable for transplantation (non-failing). Five tissues from each group were analyzed. Approximately 20 mg aliquots were embedded in an optimal cutting temperature (OCT) compound (item 62550, EMS) and stored at -80°C. 10 μm sections were mounted onto positively charged slides and either stored unfixed frozen at -80°C (for alkaline phosphatase activity staining); or fixed for 10 minutes on 10% neutral buffered formalin (for oil red O, alizarin red staining, and immunohistochemistry). For each sample, the area of alkaline phosphatase positive staining was measured in two high power microscopic fields (HPF), using a color threshold method in ImageJ. Alkaline phosphatase activity was expressed as average area per HPF. Co-localization of AP with coronary arteries and arterioles was confirmed by co-staining with anti-Actin α-Smooth Muscle (SMA) antibody. Replica sections were also stained with fluorescently-labeled endothelial-specific Isolectin B4 and SMA antibody to detect arterial and microvascular endothelial cells (as previously described [21]).

### Mouse strains and genotyping

Mice harboring a point mutation in the *ldlr* gene resulting in C699Y amino acid substitution (C57BL/6J-*Ldlr*^*Hlb301*^/J; WHC for “wicked high cholesterol” [22]) were obtained from The Jackson Laboratory (Bar Harbor, ME, USA; stock 5061). WHC were characterized in our laboratory in comparison with their parental strain C57BL/6J (Data Supplement). *Hprt*^*ALPL*^ knock-in mice were recently described [10]. *Hprt*^*ALPL*^ knock-in mice contain genomic sequence encoding chicken beta actin (CAG) promoter, a floxed “stop cassette” and the human *ALPL* cDNA (encoding TNAP) inserted into the *Hprt* locus on the X chromosome. Genetic background of *Hprt*^*ALPL*^ knock-in mice involved: 129P2/OlaHsd and C57BL/6. These were backcrossed to C57BL/6J-*Ldlr*^*Hlb301*^/J for two generation. Transgenic mice expressing *Cre* recombinase in endothelial cells (B6.Cg-Tg^Tek-Cre1Ywa^/J, abbreviated Tie2-Cre [23]) were obtained from The Jackson Laboratory (Bar Harbor, ME, USA; stock 8863). Tie2-Cre was combined with WHC mutation after crossing for two generations. Intercrossing 129;B6-*Ldlr*^Hlb301^;*Hprt*^*ALPL*^ mice with B6.Cg-*Ldlr*^Hlb301^;Tg^Tek-Cre1Ywa^ resulted in the excision of the stop cassette and activation of TNAP expression. Male mice that were homozygous for WHC mutation; hemizygous for *Hprt*^*ALPL*^ and either heterozygous for Tie2-Cre transgene (WHC-eTNAP) or wild type (WHC) were used in all experiments.

WHC mutation eliminated a BglI restriction enzyme site in exon 14. Therefore, these mice were genotyped by BglI restriction analysis of exon 14 PCR products as described [22]. *Hprt*^*ALPL*^ were genotyped as previously described [10]. *Cre* transgene was identified by PCR following the generic Cre protocol published online by The Jackson Laboratory.

### Animal studies

Animal studies were completed over the course of 2014–2017 at two locations, the Sanford Research (Sioux Falls, SD) and the New York Institute of Technology College of Osteopathic Medicine (NYIT-COM, Old Westbury, NY). Both facilities utilized 12h:12h day:night light cycle and controlled temperature and humidity, but neither was a barrier facility. Sanford Research utilized micro-isolator cages and acidified drinking water, whereas NYIT-COM facilities use conventional caging and tap water. Atherogenic diets were obtained from the same manufacturer–Teklad Diets (Envigo, Madison WI).

The initial characterization of WHC and WHC-eTNAP mice were carried out at Sanford Research. Animals were fed a standard chow or Paiden’s diet containing 42% calories from fat and supplemented with 1.25% cholesterol plus 0.5% cholate (TD.02028). Data were collected at 8 weeks of age (baseline for an atherogenic diet) and at two later time-points, 13 or 16 weeks of age.

Two additional experiments were conducted at NYIT-COM. The first experiment tested the effect of a cholate-free atherogenic diet (TD94059). This diet contained 37% of calories from fat (half of which was from cocoa butter) and was supplemented with 1.25% cholesterol. The second experiment was performed using the original Paigen’s diet (TD.02028) and tested the effect of TNAP inhibition by SBI-425 on survival, heart function, and coronary atherosclerosis.

### SBI-425 inhibitor treatment

SBI-425 was synthetized at the Prebys Center for Drug Discovery, Sanford Burnham Prebys Medical Discovery Institute (La Jolla, CA) as previously reported [10]. The inhibitor was given with powdered food at the dose of 30 mg/kg per day. Food was replenished thrice weekly. Powdered diet without active drug served as a placebo control. Treatments were initiated at 8 weeks of age and continued for five weeks. Alkaline phosphatase activity was measured in non-fasted plasma collected from the tail vein 1–3 weeks after initiation of the treatments. Cardiac function was assessed by echocardiography at baseline and at 13 weeks of age. Plasma cholesterol, triglycerides, calcium, and inorganic phosphate were measured in fasted plasma. Coronary calcification and atherosclerosis were analyzed histologically.

### Echocardiography

Sanford Research animal facility provided access to Vevo2100 ultrasound imaging system (FUJIFILM Visual Sonics, Toronto, Canada) equipped with MS400 and MS550D transducers (18–38 and 22–55 MHz); NYIT-COM provided access to Vivid 7 ultrasound instrument (GE Healthcare, Port Washington, NY) equipped with i13L transducer recommended for cardiac studies in rodents (5.9–14.1 MHz). Mice were anesthetized with 0.8–1.5% isoflurane and a parasternal short axis view was obtained in B-mode and recorded in M-mode. The M-mode echocardiograms were analyzed by tracing myocardial wall movement over 3–5 cardiac cycles using Vivo2100 image analysis software. Alternatively, left ventricular (LV) diameter, LV posterior and septal wall thicknesses in systole and diastole were recorded with time intervals for three cardiac cycles using EchoPac software (GE Healthcare, Port Washington, NY). Heart rate, ejection fraction, cardiac output, and LV mass were calculated using standard equations for rodents (as recommended by Visual Sonics).

### Blood chemistry

Heparin plasma was prepared from venous blood collected from the right ventricle of anesthetized mice. Mice were fasted at least 5 hours before blood collection. Alkaline phosphatase (AP) activity, calcium, phosphorus, triglycerides, and cholesterol were determined using the ADVIA® 1800 Clinical Chemistry analyzer (Siemens, Tarrytown, NY) utilizing specific reagents: ALPAMP, CA_2, IP, TRIG, and CHOL_2. These methods were available and applied to all samples collected at Sanford Research. Inorganic pyrophosphate (PP_i_) was measured in lithium heparin plasma according to the method described previously [24]. Samples collected at the NYIT-COM were analyzed following the manual protocols in a plate reader (BioTek Instruments, Winooski, VT) operated in the kinetic (AP) or end-point mode (cholesterol, triglycerides, calcium, and phosphorus). PP_i_ concentration in those samples were determined based on luminescent detection of the ATP product of an ATP sulfurylase reaction, which uses PP_i_ as a substrate [25].

### Histology and morphometry

#### Quantification of atherosclerosis in the aortic root and coronary arteries of mice

Tissues for quantitation of atherosclerosis were prepared according to a recommended protocol [26] with some modifications: 10 μm consecutive sections were collected over 640 μm region starting from the beginning of the aortic valve cusps and included the proximal left coronary artery and the coronary artery branch in the septum. In the majority of our samples, the septal branch was supplied by the right coronary artery. This observation was consistent with the previously published anatomical report [27]. Every 8^th^ consecutive section in this region was analyzed by each staining method. Alizarin red staining was used to detect calcium; oil red O stain was used to measure plaque area (counterstained with hematoxylin). Areas of positive staining were measured in the ImageJ software (NIH, Bethesda, MD). Aortic roots and coronary arteries were outlined manually; oil red O and alizarin red-positive areas were measured within these selected non-overlapping areas by setting a color threshold. 6–8 sections per sample for the aortic root (minimum 3 per sample) and 4–6 sections per sample for the coronary arteries were analyzed and the area measurements were averaged for each mouse in each region.

#### Other histology

TNAP activity was detected in unfixed undecalcified human and mouse tissues. These were embedded in OCT; 10 μm cryosections were prepared mounted on positively-charged slides and either immediately processed or stored frozen at -80°C before the analysis. Slides were warmed up to room temperature; BCIP/NBT alkaline phosphatase substrate (item S3771, Promega) was applied directly onto the tissue sections and incubated for 5 minutes at room temperature. Slides were rinsed in water, post-fixed in 10% neutral buffered formalin for 10 minutes at room temperature, rinsed in distilled water, dehydrated in 95% ethanol, air-dried, and mounted in Clearium Mounting Media (item 13520, EMS). To determine if the tissue activity can be attributed to TNAP, a specific inhibitor L-homoarginine (item H1007, Sigma) was added to the substrate mixture at final concentration of 12.5 mM [28]. Human tissues, used for the detection and vascular co-localization of AP activity, were described above (see Human Myocardial Samples). Mouse tissues were analyzed at baseline (hearts and aortic roots) and at 16 weeks of age on a Paigen’s diet (a panel of tissues consisting of heart, aortic root, aorta, lung, and mesentery; one representative sample per group).

Aortic root samples from a subset of 13 and 16-weeks-old WHC and WHC-eTNAP mice with atherosclerosis (n = 3/group) were embedded in paraffin and processed by H&E staining (for general pathology) or picrosirius red staining (to visualize collagen).

Osteocalcin was visualized by immunohistochemistry in the aortic root sections from 16-weeks old mice (n = 2/group) using rabbit polyclonal antibody (item M173; Tanaka Bio Inc) followed by Alexa 488–labeled anti‐rabbit IgG secondary antibody (item 711‐546‐152; Jackson ImmunoResearch).

Arteries in human myocardial tissues were detected by immunohistochemistry. We used anybody specific to α-Smooth Muscle Actin (SMA), which was directly labeled with horse radish peroxidase, HRP, at 1:200 dilution (clone 1A4, Item sc-32251, Santa Cruz Biotechnology). Sections were incubated with AP substrate first; washed in water; fixed in formalin for 10 minutes at room temperature; treated with BLOXALL reagent to stop endogenous AP and peroxidase activities (item SP-6000, Vector Labs) as recommended; washed in PBS; and incubated with SMA-HRP antibody for one hour at room temperature. After washing in PBS, HRP activity was detected using ImmPACT DAB Peroxidase Substrate (item SK-4105, Vector Lab). Slides were rinsed in water, dehydrated in ethanol, air-dried, and mounted in Clearium (item 13520, EMS).

### Real time PCR

RNA was isolated from the aortas using a Maxwell 16LEV simplyRNA Tissue kit (item AS1280; Promega). RNA quantity and quality were determined using a NanoDrop 1000 (Thermo Scientific). Equal amounts of RNA from each sample were converted to cDNA using GoScript reverse transcriptase (item A501C; Promega) with random hexamer primers. Gene expression was evaluated using primers obtained from the Integrated DNA Technologies (*Gapdh* Mm.PT.39a.1; *Sp7* Mm.PT.58.10898265; *Bmp2* Mm.PT.58.10419414; *Runx2* Mm.PT.58.41866893; *Sox9* Mm.PT.58.42739087; *Bglap* Mm.PT.58.9119501.g; *Spp1* Mm.PT.56.43709208; *Mgp* Mm.PT.58.32674923; *Acan* Mm.PT.58.10174685; *Col2a1* Mm.PT.58.41487458; *Opg* Mm.PT.56a.414946; *Tnfsf11* Mm.PT.55a.29202697) and SYBR green polymerase master mix from QIAGEN. Gene expression was normalized to *Gapdh* and expressed as arbitrary units calculated according to the deltaCt method.

### Statistics

GraphPad Prism version 7 (GraphPad Software) was used for all analyses. Data were expressed as mean ± standard deviation (SD). The F tests or Brown-Forsythe tests were used to compare variances. The parametric or non-parametric tests were applied as appropriate depending on if variances were equal or unequal. The Student's t-test or Mann-Whitney U test was used to compare means between two groups. The one-way ANOVA or Kruskal-Wallis tests were computed to compare multiple groups. In cases where an overall p value for the ANOVA or Kruskal-Wallis models was significant, we performed the selected pairwise group comparisons (p values for these comparisons were adjusted for multiple comparisons according to Bonferroni's or Dunn's methods). Groups that differed by the diet, genotype, or treatment were compared. All analyses were pre-specified and calculated for each age separately. No changes from baseline were analyzed in this study. Survival curves were compared by log-rank Mantel-Cox test. The significance was accepted at p < 0.05.

## Results

### TNAP activity in human myocardium

**Fig 1:**
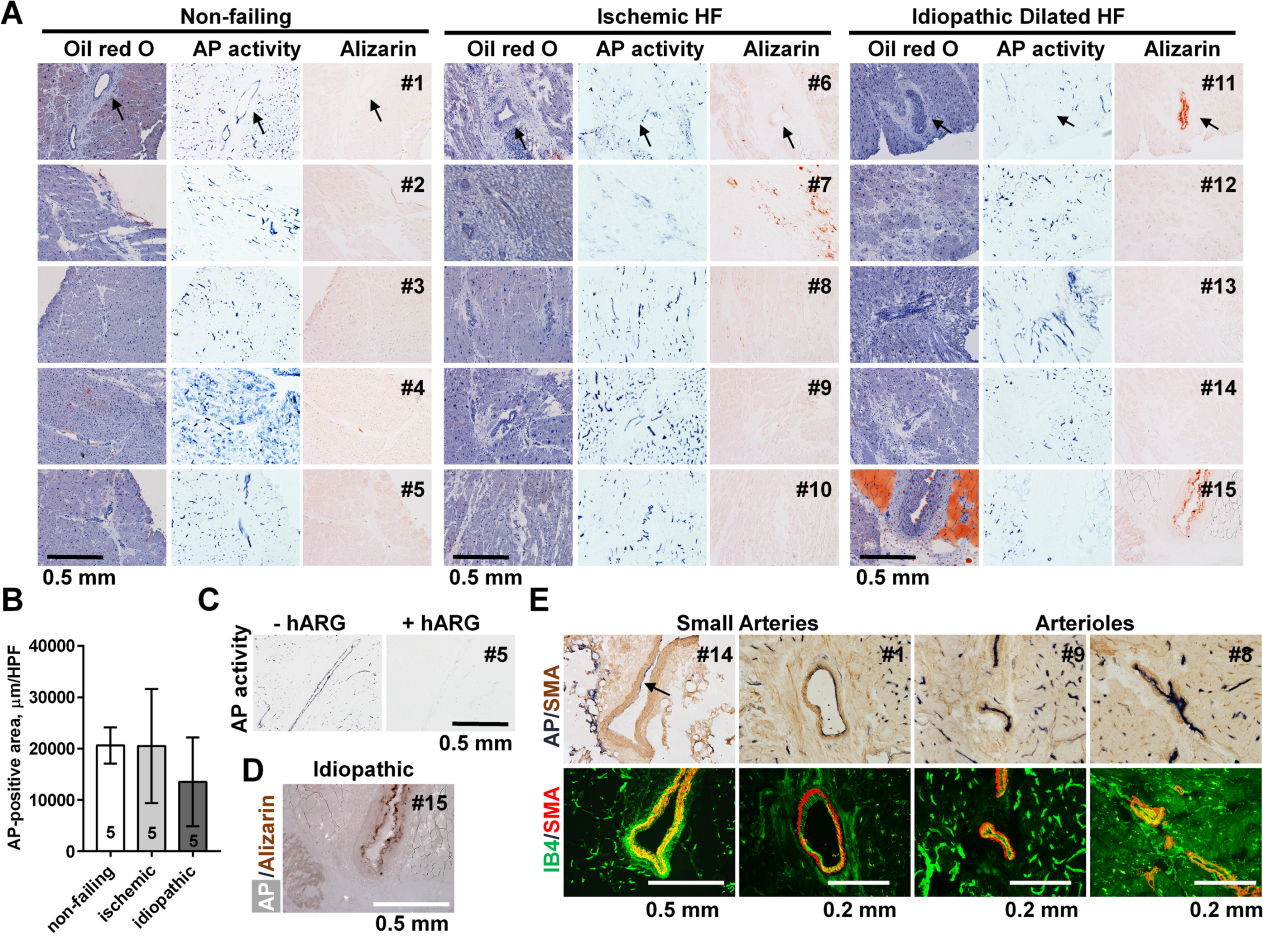
Endothelial TNAP activity in human myocardium. **(A)** Fifteen samples were analyzed—five from non-failing donor hearts (#1–5); five from ischemic HF (#6–10), and five from idiopathic dilated HF patients (#11–15); serial sections from each sample were stained with oil red O (hematoxylin counterstain), AP activity, and alizarin red; microscopic fields containing arteries were identified and captured; arrows, small arteries. **(B)** Quantification of AP activity in each category of samples expressed as AP-positive area per high power field (HPF). **(C)** Representative consecutive sections from sample #5 (non-failing) stained for AP activity in the absence (left) or presence (right) of 12.5 mM of L-homoarginine, a specific TNAP inhibitor. **(D)** A photomerged overlay image of AP activity (white) and alizarin red staining (brown) from sample #15 (idiopathic dilated HF); **(E)** Top panels, AP activity (dark blue) in combination with α-smooth muscle actin (SMA) immunohistochemistry (brown); bottom panels, same vessels stained with SMA antibody (red) in combination with endothelial specific isolectin B4 (IB4, green); arrow, AP activity.

It has been long recognized that alkaline phosphatase (AP) activity is expressed in the endothelial cells of terminal arterial tree in multiple tissues in experimental animals [29]. AP activity was also found in the endothelium of larger arteries in the skeletal muscle of birds and mammals including mouse, guinea pig, dog, monkey, and human [30]. Less is known about activity of endothelial TNAP in human myocardium in different disease states [31]. Here we analyzed AP activity in the myocardial vasculature in five rejected non-failing transplant donor hearts and ten failing hearts obtained from cardiac transplantation—five ischemic and five idiopathic dilated heart failure (HF). Lipids were not detected in the coronary arteries captured in any of the samples (oil red O staining, **Fig 1A**). Alkaline phosphatase (AP) activity was detected in all samples and anatomically co-localized with the vasculature; both micro-vessels and small arteries contained activity (AP activity, **Fig 1A**). No differences in average areas of AP-positive staining were noted between non-failing and failing hearts (non-failing: 20624 ± 3525 μm/HPF; ischemic: 20510 ± 11132 μm/HPF; idiopathic 13520 ± 8646 μm/HPF; ANOVA p = 0.34; **Fig 1B**). In every sample, AP activity was sensitive to inhibition by 12.5 mM L-homoarginine, a specific TNAP inhibitor [28] (an example is shown in **Fig 1C**). Vascular calcification was detected in all three groups: 2/5 non-failing; 3/5 of ischemic; and 2/5 idiopathic dilated HF samples were positive for alizarin red staining; the staining was anatomically co-localized with the arteries (alizarin red, **Fig 1A**). In one ischemic HF sample calcification was also detected within myocardial scar tissue. In one idiopathic dilated HF sample TNAP activity and micro-calcification co-localized with the luminal side of a small artery. In this sample, calcification appeared to be present in the internal elastic lamina of the affected vessel (**Fig 1D**). We confirmed vascular localization of TNAP activity by showing that it is associated with the luminal side of small coronary arteries and arterioles detected by smooth muscle actin (SMA) immunohistochemistry. In another set of sections SMA antibody was used in combination with endothelial specific lectin-based fluorescent staining showing endothelial luminal lining of these vessels as well as microvascular staining. These assays were performed on all 15 samples (confirming our morphologic definitions of arteries in **Fig 1A**) and representative images are shown in **Fig 1E**. We noted that endothelial TNAP is more active in arterioles, but it is also present in the endothelium of small arteries. No larger arteries were present in our samples.

### An empirical hypothesis and the experimental model

**Fig 2:**
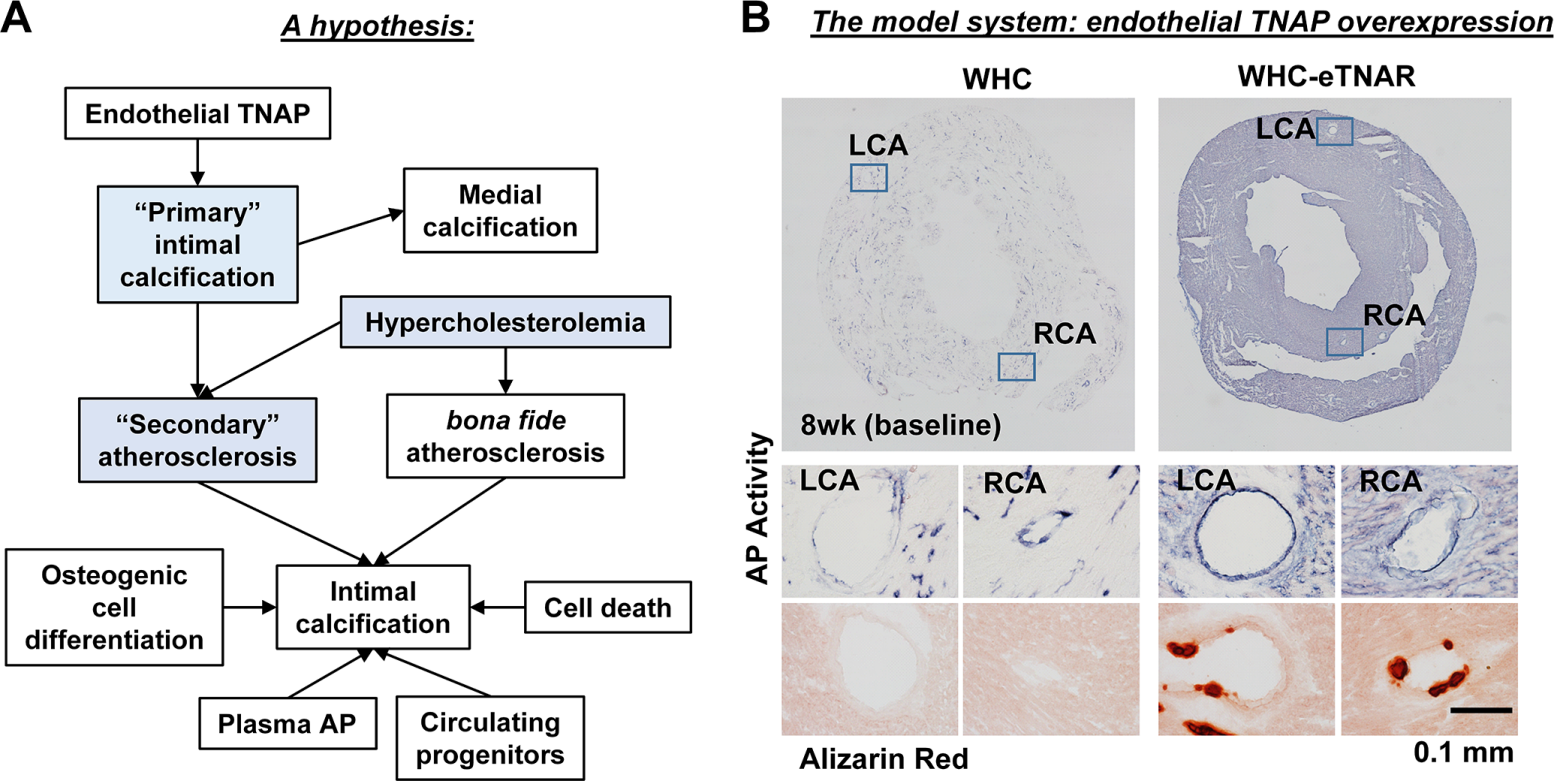
A working hypothesis and the experimental model. **(A)** Based on existing evidence from our animal model, we postulated that upregulated activity of endothelial TNAP can increase intimal calcification prior to development of atherosclerosis; “primary” calcified lesions in the intima can serve as focal points for lipid deposition or “secondary” atherosclerosis by increasing endothelial roughness. This type of accelerated lipid deposition might take place in coronary arteries of WHC-eTNAP mice that are rendered hypercholesterolemic by an atherogenic diet. Components of the model that are being tested in this study are highlighted in blue. **(B)** AP activity staining in the hearts of WHC and WHC-eTNAP mice and detection of vascular calcification in WHC-eTNAP mice at baseline (8 weeks of age) in coronary arteries prior to induction of hypercholesterolemia.

Previously, we have demonstrated that mice with transgenic overexpression of TNAP in endothelial cells (eTNAP mice) develop an adult-onset vascular calcification detectable in the coronary arteries by 8 weeks of age [11]. In the present experiment, we wished to test a hypothesis that such “primary” intimal calcification might create a permissive environment for the “secondary” atherosclerotic plaque formation (**Fig 2A**). This hypothetical scenario is a research idea that is grounded in our empirical observations but contradicts the classical pathophysiologic mechanism of atherogenesis, which describes intimal calcification as a process that occurs only at the later stages of plaque formation [32]. To test our hypothesis, TNAP was overexpressed in endothelium of mice harboring a point mutation in the low density lipoprotein receptor, *ldlr*^*hbl301*^ (also known as “wicked high cholesterol” mice [22], abbreviated here WHC). As expected, WHC-eTNAP mice displayed upregulated AP activity in endothelial cells and formation of sub-endothelial calcific nodules in coronary arteries detected by alizarin red staining at baseline (**Fig 2B**).

We selected the WHC mutation as our basic model of hypercholesterolemia because it recapitulates one of the disease-causing mutations in human familial hypercholesterolemia at the genetic level [22]. It is an interesting model because the mutation is amenable to genome editing, an attractive future therapeutic goal. Our own characterization of WHC mice is detailed in the Supplement. We are providing evidence that this model recapitulates all basic aspects of murine atherosclerosis, including hypercholesterolemia (**S1A and S1B Fig**), aortic root atherosclerosis (**S1C Fig**); typical morphology of plaques in the aortic root, including calcifications in advanced lesions (**S1D Fig**); and relatively normal left ventricular function (**S1 Table**). We have also demonstrated endogenous AP activity in WHC mice in association with aortic plaque calcifications (**S1D Fig**); and in vasculature, including coronary arteries (**S1E Fig**). Detection of endogenous alkaline phosphates (AP) activity in the vasculature was not unique to WHC as we have observed similar distribution AP activity in control animals without the WHC mutation or atherosclerosis [11]. It was reported that WHC mice can develop coronary atherosclerosis [22]; we have found a low incidence of plaque formation in coronary arteries of WHC mice; the coronary plaques that were found in this model were located in the coronary ostia (**S1D Fig,** arrow), whereas proximal coronary arteries were always spared under the conditions or our experiments (**S1E Fig**). We examined all coronary arteries in all samples at 23 weeks of age and did not find any areas positive for alizarin red staining (data not shown). Therefore, we have sufficiently established that the WHC model will provide an appropriate background for testing our hypothesis regarding TNAP overexpression, calcification, and atherosclerosis.

### Effects of an atherogenic diet in WHC and WHC-eTNAP mice

Starting at 8 weeks of age (baseline), WHC and WHC-eTNAP mice were switched to an atherogenic diet (Paigen’s type) or continued on a standard diet. They were followed until 13 weeks of age (5 weeks on a diet) or 16 weeks of age (8 weeks on a diet). 16 weeks of age corresponded to the median survival age of WHC-eTNAP mice on an atherogenic diet determined in a pilot study (data not shown). Increased mortality of WHC-eTNAP mice on an atherogenic diet (but not on a control diet) could be attributed in part to peripheral atherosclerosis, a topic outside of the scope of our current report.

#### Blood chemistry

Blood chemistry was analyzed in fasted plasma, the results are tabulated in **Table 1**. As expected, we found significant upregulation of plasma alkaline phosphatase activity in WHT-eTNAP compared to WHC (baseline: 3142 vs. 93 mU/L, p<0.001; 13wk control diet: 3138 vs. 85 mU/L, p<0.01; 13wk athero diet: 3557 vs. 95 mU/L, p<0.01; 16wk control diet: 1908 vs. 47 mU/L, p<0.0001; 16wk athero diet: 3654 vs. 121 mU/L, p<0.01). At 16 weeks of age, the activity in WHT-eTNAP on an athero diet was higher than on a control diet (p<0.0001). This last observation is interesting because it suggests that plasma TNAP activity or TNAP vesicular shedding might be regulated by an interaction between age and diet, however this single highly significant observation might has also occurred by chance considering the large number of parameters investigated. Calcium and phosphorus levels were not significantly different between groups in this study except for 16-weeks-old WHC on an athero diet, in which calcium and phosphorus levels were lower compared to a control diet (Ca: 8.7 vs 9.2 mg/dl, p<0.05; Pi: 5.6 vs 8.3 mg/dl, p<0.05). Triglycerides (TG) were slightly elevated in WHC-eTNAP compared to WHC at baseline (123 vs. 81 mg/dl, p<0.01); TG were also increased in 13-weeks-old WHC on an athero diet compared to a control diet (311 vs. 89 mg/dl, p<0.05). Both WHC and WHT-eTNAP mice had elevated plasma cholesterol on an athero diet compared to a control diet (13wk WHC: 3054 vs. 273, p<0.001; 13wk WHC-eTNAP: 2913 vs. 358, p<0.05; 16wk WHC: 2889 vs. 263, p<0.05; 16wk WHC-eTNAP: 3019 vs. 316, p = 0.08). Pyrophosphate (PP_i_) was measured at 16 weeks of age. PP_i_ levels were higher in mice on an athero diet compared to a control diet (WHC: 4.9 vs.1.6 μM, p<0.01; WHC-eTNAP: 4.7 vs. 0.7 μM, p<0.001). TNAP overexpression had no effect on plasma PP_i_ in this model, which was consistent with our published data obtained in the endothelial TNAP model in the absence of the WHC mutation [11].

**Table 1:** Blood chemistry of WHC and WHC-eTNAP mice (Mean ± SD).

		Control diet		Athero diet (Paigen’s)	
Parameter	Age group	WHC	WHC-eTNAP	WHC	WHC-eTNAP
N	baseline	6	9		
	13 wk	9	7	6	8
	16 wk	4	5	4	5
AP, mU/L	baseline	93 ± 11	3142 ± 429***		
	13 wk	85 ± 14	3138 ± 518**	95 ± 21	3557 ± 479**
	16 wk	47 ± 7	1908 ± 226****	121 ± 27	3654 ± 661**** ††††
Ca, mg/dl	baseline	9.0 ± 0.3	9.3 ± 0.5		
	13 wk	9.4 ± 0.8	9.2 ± 0.5	8.7 ± 0.3	8.5 ± 0.6
	16 wk	9.2 ± 0.2	9.2 ± 0.1	8.7 ± 0.1†	9.2 ± 0.3*
P_i_, mg/dl	baseline	5.4 ± 0.4	5.6 ± 1.0		
	13 wk	6.8 ± 2.2	6.8 ± 1.5	5.6 ± 1.1	7.5 ± 3.3
	16 wk	8.3 ± 1.4	7.8 ± 1.1	5.6 ± 0.6†	6.8 ± 0.6
TG, mg/dl	baseline	81 ± 17	123 ± 29**		
	13 wk	89 ± 51^(n = 8)^	105 ± 48	311 ± 97†	159 ± 124
	16 wk	106 ± 32	116 ± 44	139 ± 58	131 ± 33
CHOL, mg/dl	baseline	331 ± 56	343 ± 64		
	13 wk	273 ± 81^(n = 8)^	358 ± 62	3054 ± 466†††	2913 ± 620†
	16 wk	263 ± 26	316 ± 42	2889 ± 74†	3019 ± 405
PP_i_, mg/dl	16wk	4.9 ± 1.7^(n = 4)^	4.7 ± 1.5^(n = 5)^	1.6 ± 0.8^(n = 4)^ ††	0.7 ± 0.5^(n = 5)^ †††

AP, alkaline phosphatase; Ca, calcium; P_i_, phosphorus; TG, triglycerides; CHOL, cholesterol; PP_i_, pyrophosphate

* p < 0.05

** p < 0.01

*** p < 0.001

**** p < 0.0001 vs. WHC of the same age and diet

† p < 0.05

†† p < 0.01

††† p < 0.001

†††† p < 0.0001 vs. the control diet group of the same age and genotype

#### Physiology

Physiology data are summarized in **Table 2**. WHC and WHC-eTNAP mice on an atherogenic diet were smaller compared to a control diet; the differences in body weight were greater and manifested earlier in WHC-eTNAP (16wk WHC: 23.6 vs. 29.0 g, p<0.01; 13wk WHC-eTNAP: 23.6 vs. 25.9 g, p<0.01; 16wk WHC-eTNAP: 19.5 vs. 26.6 g, p<0.001). At 16 weeks of age, WHC-eTNAP on an athero were also smaller than WHC on the same diet (p<0.01). At 16 weeks of age, WHT-eTNAP on an athero were had lower ejection fraction (EF) than WHC on the same diet (35 vs 47%, p<0.05); the EF in WHT-eTNAP was lower on an athero diet when comparing to a control diet (31 vs. 53%, p<0.05). At 16 weeks of age, both WHC and WHC-eTNAP mice had reduced cardiac output (normalized to BW) on an athero diet compared to a control diet (WHC: 0.50 vs. 0.63 ml*min^-1^*g^-1^, p<0.01; WHC-eTNAP: 0.44 vs. 0.63 ml*min^-1^*g^-1^, p<0.01).

**Table 2:** Physiological characteristics of WHC and WHC-eTNAP mice (Mean ± SD).

		Control diet		Athero diet (Paigen’s)	
Parameter	Age group	WHC	WHC-eTNAP	WHC	WHC-eTNAP
N	baseline	30	33		
	13 wk	17	13	14	16
	16 wk	4	4	7	7
BW, g	baseline	24.9 ± 2.6	23.7 ± 2.5		
	13 wk	27.2 ± 2.1	25.9 ± 2.8	25.3 ± 2.2	23.6 ± 2.1†
	16 wk	29.0 ± 2.6	26.6 ± 1.7	23.6 ± 1.1††	19.5 ± 2.6**†††
HR, bpm	baseline	436 ± 28	429 ± 33		
	13 wk	426 ± 40	424 ± 46	449 ± 49	451 ± 49
	16 wk	460 ± 36	424 ± 13	399 ± 38†	379 ± 28
LV EDD, mm	baseline	3.9 ± 0.2	3.9 ± 0.2		
	13 wk	3.9 ± 0.3	3.8 ± 0.3	3.8 ± 0.3	3.8 ± 0.2
	16 wk	4.0 ± 0.1	4.1 ± 0.3	3.8 ± 0.5	3.5 ± 0.5
EF, %	baseline	55 ± 5	55 ± 6		
	13 wk	57 ± 7	57 ± 7	57 ± 7	53 ± 9
	16 wk	58 ± 4	53 ± 5	47 ± 3	35 ± 11*††
CO/BW, ml*min^-1^*g^-1^	baseline	0.63 ± 0.09	0.65 ± 0.10		
	13 wk	0.57 ± 0.09	0.57 ± 0.09	0.62 ± 0.08	0.61 ± 0.12
	16 wk	0.63 ± 0.02	0.61 ± 0.04	0.50 ± 0.07†	0.44 ± 0.10†
LV mass/BW, mg*g^-1^	baseline	3.2 ± 0.4	3.3 ± 0.5		
	13 wk	3.1 ± 0.4	3.3 ± 0.3	2.9 ± 0.4	3.6 ± 0.9
	16 wk	3.0 ± 0.1	3.4 ± 0.4	2.7 ± 0.2	3.4 ± 1.1

BW, body weight; HR, heart rate; LV EDD, end diastolic diameter of the left ventricle; EF, ejection fraction; CO, cardiac output; LV mass, left ventricular mass

* p < 0.05

** p < 0.01, WHC-eTNAP vs. WHC of the same age and diet

† p < 0.05

†† p < 0.01

††† p < 0.001, atherogenic vs. the same age and genotype on a control diet

#### Effect of TNAP overexpression on coronary and aortic root atherosclerosis

Our previous study of eTNAP mice (in the absence of hypercholesterolemia) indicated that different vascular beds were differentially affected by calcification and that small and medium-sized arteries (such as coronary and mesenteric) were preferentially affected compared to the aorta [11]. We hypothesized that “primary” intimal calcification in the coronary arteries of WHC-eTNAP mice (**Fig 2**) will accelerate lipid deposition, whereas aortic root region that is unaffected by calcification at baseline will display normal course of atherosclerosis resulting in a *bona fide* atherosclerotic plaques. We have also suspected that plagues in the aortic root of WHC-eTNAP mice might become more calcified, in part because of increased alkaline phosphatase activity in plasma.

**Fig 3:**
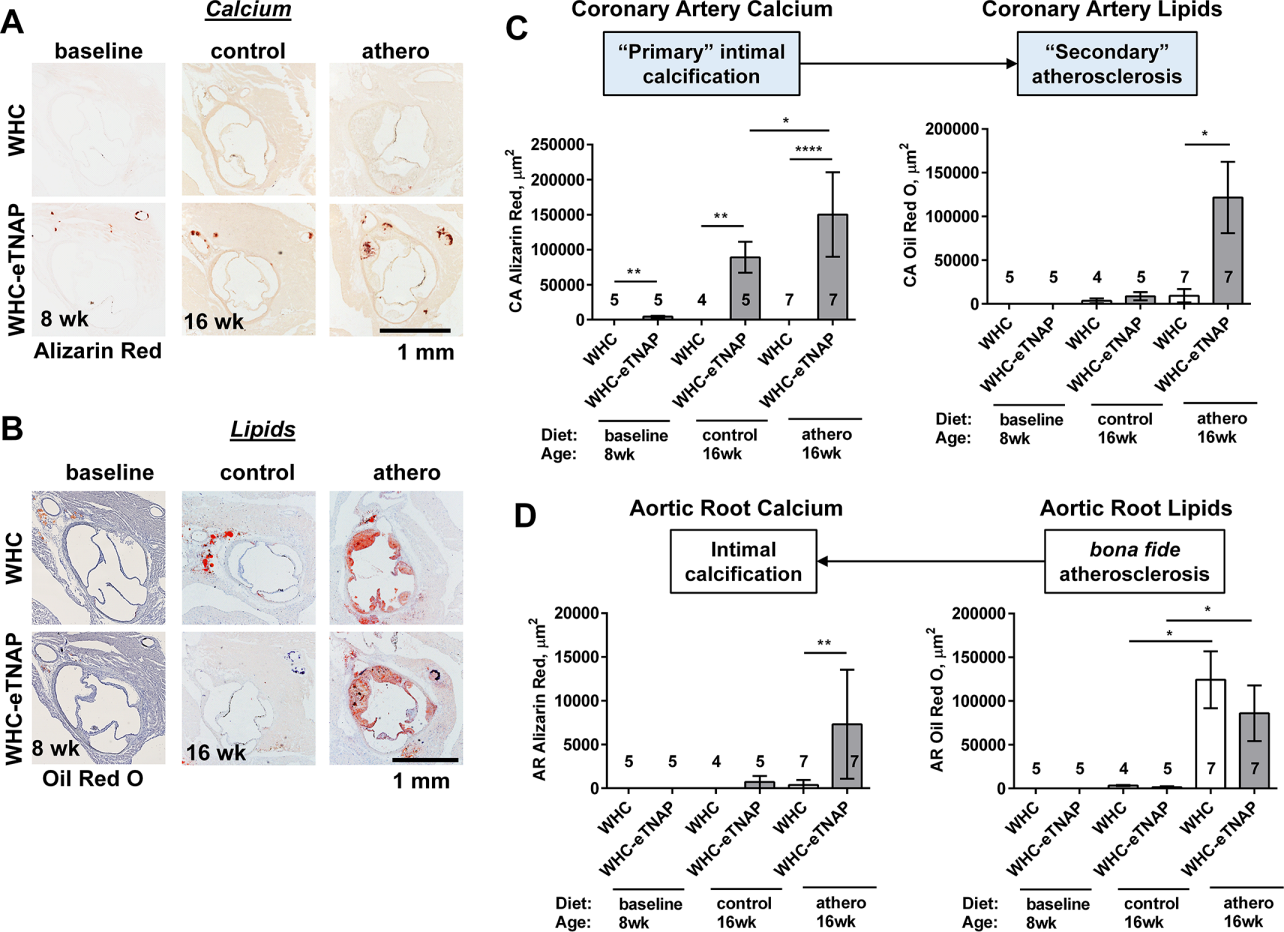
Atherosclerosis in the coronary arteries and the aortic root of WHC and WHC-eTNAP mice. **(A)** Alizarin red staining for calcium, representative images. **(B)** Oil red O staining, hematoxylin counterstained; representative images. **(C)** Quantification of calcium (based on alizarin red staining) and lipids (oil red O staining) in the coronary arteries indicates that, under conditions of our model, calcification in the coronary arteries precedes and might be promoting lipid deposition. **(D)** Quantification of calcium (alizarin red staining) and lipids (oil red O staining) in the aortic root indicates that, under conditions of this experiment, calcification follows lipid deposition. All data were collected at baseline and at 16 weeks of age. *, p < 0.05; **, p < 0.01; ****, p < 0.0001.

Coronary artery calcification was measured as area of positive alizarin red staining in serial sections of the aortic root (**Fig 3A**). Coronary artery lipid deposition was measured as area of positive Oil Red O staining in serial sections of the aortic root (**Fig 3B**). Coronary artery calcification was absent in WHC mice and significantly increased in WHC-eTNAP mice at baseline and at 16 weeks of age on both diet—control and atherogenic (baseline: 4312 vs. 0 μm^2^, p<0.01; 16wk control diet: 89214 vs. 0 μm^2^, p<0.01; 16wk athero diet: 150152 vs. 0 μm^2^, p<0.001; **Fig 3C,** left). Coronary calcium was also found to be higher in WHC-eTNAP mice on an athero diet compared to a control diet (p<0.05). Coronary artery lipids were not detected at baseline; detection of lipids was significantly increased in the coronary arteries of 16-weeks-old WHC-eTNAP mice on an athero diet compared to WHC mice on the same diet (121633 vs. 9330 μm^2^, p<0.05; **Fig 3C**, right). There was an increase in calcification of the plaques in the aortic roots of 16-weeks-old WHC-eTNAP mice on an athero diet compared to WHC on the same diet (7305 vs. 371 μm^2^, p<0.05; **Fig 3D**, left), whereas oil red O-positive plaque areas in the aortic root were not different between WHC-eTNAP and WHC under these conditions (85942 vs. 124237 μm^2^, **Fig 3D**, right).

Alkaline phosphatase (AP) activity was examined in histological sections from a panel of tissues from 16-weeks-old WHC and WHC-eTNAP mice with atherosclerosis (**S2 Fig**). Endogenous AP activity was detected in the aortic root sections of WHC, where it was more prominent in the adventitia and absent in the plaque (**S2A and S2B Fig**, left). WHC mice exhibited low activity in the descending thoracic aorta (**S2C Fig**, left). Endogenous AP activity was also detected in medial layer of the mesenteric arteries of WHC mice (**S2D Fig**, left) and in the lungs (**S2E Fig**, left). AP activity was highly upregulated in WHC-eTNAP mice compared to WHC; it was detected in plaques in the coronary artery ostium and aortic sinus (**S2A and S2B Fig**, right); in the medial layer in the aortic root and descending aorta (**S2B and S2C Fig**, right); in mesenteric arteries and surrounding tissue (**S2D Fig**, right); and in the lung (**S2E Fig**, right).

It has been suggested by a recent study that secondary calciprotein particles (CPP) secreted by calcifying cells can induce release of TNFα *in vitro* [33]. It is plausible that TNAP-driven calcification in our *in vivo* model might also increase secretion of CPP and promote inflammation *via* upregulation of TNFα that in turn could explain the excess of coronary atherosclerosis in WHC-eTNAP mice. Moreover extracellular particles, including calcium phosphate crystals, can induce inflammasome activation in macrophages and stimulate the release of IL-1β [34]. To test these suggestions, we have measured circulating levels of TNFα and IL-1β in plasma of WHC and WHC-eTNAP mice at 13 and 16 weeks of age on control and atherogenic diets. There was no difference detected between two age groups in the expression of either cytokine, therefore we pooled the data to increase the power of analysis. The results are shown in **S3 Fig**. TNFα was increased in both WHC and WHC-eTNAP on an athero diet compared to a control diet (WHC: 4.0 vs. 1.5 pg/ml, p<0.05; WHC-eTNAP: 5.5 vs. 0.3 pg/ml, p<0.05); we detected no statistically significant differences in TNFα detected between WHC and WHC-eTNAP groups on either control or an atherogenic diet (**S3A Fig**). Concentrations of IL-1β in plasma were also not different between the groups. IL-1β values were as follows: WHC control diet—3.1 pg/ml; WHC-eTNAP control diet—1.0 pg/ml; WHC athero diet—0.2 pg/ml; WHC-eTNAP athero diet—7.9 pg/ml (**S3B Fig**). There was on outlier in WHC-eTNAP athero diet group that had higher levels of both cytokines: TNFα - 31.9 pg/ml; IL-1β– 51.7 pg/ml. We concluded that CPP and inflammation might play a role in this condition but requires further investigation.

To rule out the possibility that pro-inflammatory [35] or other special pro-atherosclerogenic properties of cholate [36] in Paigen’s diet are responsible for an unusual course of coronary artery disease in our model, we repeated our experiment using a cholate-free diet containing the same amount of cholesterol (TD94056). Dietary treatment was initiated at 8 weeks of age and atherosclerosis was analyzed at 13 weeks of age. Both coronary calcium and lipid deposition were increased in WHC-eTNAP mice compared to WHC (calcium: 38985 vs. 68 μm^2^, p = 0.06; lipids: 16780 vs. 280 μm^2^, p = 0.06; **S4 Fig**). Lipids in aortic root plaques were not different (WHC: 22637 μm^2^; WHC-eTNAP: 37771 μm^2^); and no calcifications were detected in aortic root plaques at this time point. No differences in physiologic parameters were noted between the two groups; except for reduced body weight in WHC-eTNAP mice compared to WHC at 13 weeks of age (24.7 vs 29.7 g, p<0.05; **S2 Table**). We concluded that accelerated coronary atherosclerosis in WHC-eTNAP mice is not caused by cholate in Paigen’s diet.

#### Gene expression

The expression of osteogenic and chondrogenic markers was examined next. This was performed using mRNA isolated from descending thoracic aortas of 16-weeks-old mice (**Table 3**). In contrast to our previous studies [10, 11], we found no effect of TNAP overexpression on *Runx2* expression. We repeated *Runx2* qPCR in 13-weeks-old mice with similar negative results (**S5A Fig**). Runx2 immunohistochemistry was performed on aortic roots and calcified coronary lesions from 8-weeks-old mice and also failed to detect differences between WHC and WHC-eTNAP (**S5B Fig**). The lack of upregulation of *Runx2* expression in this study might be partly due to differences in genetic background. Our previous studies were performed on a mixed 129.B6 background. For this experiment, TNAP transgene was backcrossed to C57BL/6-WHC for two generation resulting in a greater C57BL/6 allele pool. The lack of *Runx2* upregulation could also be linked to downregulation of Bmp2 expression [37]. The O*sterix* and *bone morphogenic protein 2* (*bmp2)* mRNAs were downregulated in WHC-eTNAP aortas compared to WHC, both under atherogenic conditions (*osterix*: 1.0 vs. 2.7 AU, p<0.01; *bmp2*: 48 vs. 95 AU, p<0.05). *Osteopontin* expression was upregulated in the aortas of WHC-eTNAP under atherogenic conditions compared to a control diet (2845 vs. 866 AU, p<0.05). Expression of *matrix gla protein (mgp)* and *osteoprotegerin* (*opg)* mRNAs was increased in the aortas of WHC on an athero diet compared to a control diet (*mgp*: 12882 vs. 6552 AU, p < 0.05; *opg*: 112 vs. 50 AU, p<0.01).

**Table 3:** Expression of osteogenic and chondrogenic markers in aortas of WHC and WHC-eTNAP mice at 16 weeks of age (arbitrary units; Mean ± SD).

	Control diet		Athero diet (Paigen’s)	
Parameter	WHC	WHC-eTNAP	WHC	WHC-eTNAP
N	3	4	7	7
*Runx2*	36 ± 14	28 ± 4	48 ± 16	47 ± 31
*Osterix*	2.1 ± 0.7	1.3 ± 0.5	2.7 ± 1.2	1.0 ± 0.1**
*Bmp2*	57 ± 13	50 ± 3	95 ± 48	48 ± 13*
*Osteocalcin*	1.9 ± 1.2	1.8 ± 0.3	2.0 ± 0.6	1.9 ± 1.3
*Osteopontin*	3.0 ± 1.3	54 ± 32	866 ± 505	2845 ± 1851†
*Mgp*	6552 ± 3108	6818 ± 663	12882 ± 3482†	9220 ± 3374
*Opg*	51 ± 23	50 ± 9	112 ± 27††	89 ± 27
*Rankl (x1000)*	3.4 ± 3.3	1.9 ± 0.4	5.4 ± 3.8	9.3 ± 9.4
*Sox9*	30 ± 16	26 ± 8	49 ± 10	48 ± 19
*Aggrecan*	130 ± 60	112 ± 25	161 ± 52	93 ± 55
*Col2a1*	0.13 ± 0.06	1.46 ± 2.01	0.36 ± 0.27	1.39 ± 2.48

* p < 0.05

** p < 0.01, WHC-eTNAP vs. WHC on the same diet

† p < 0.05

†† p < 0.01, atherogenic vs. control diet in mice of the same genotype

### Histology of coronary lesions in WHC-eTNAP mice

**Fig 4:**
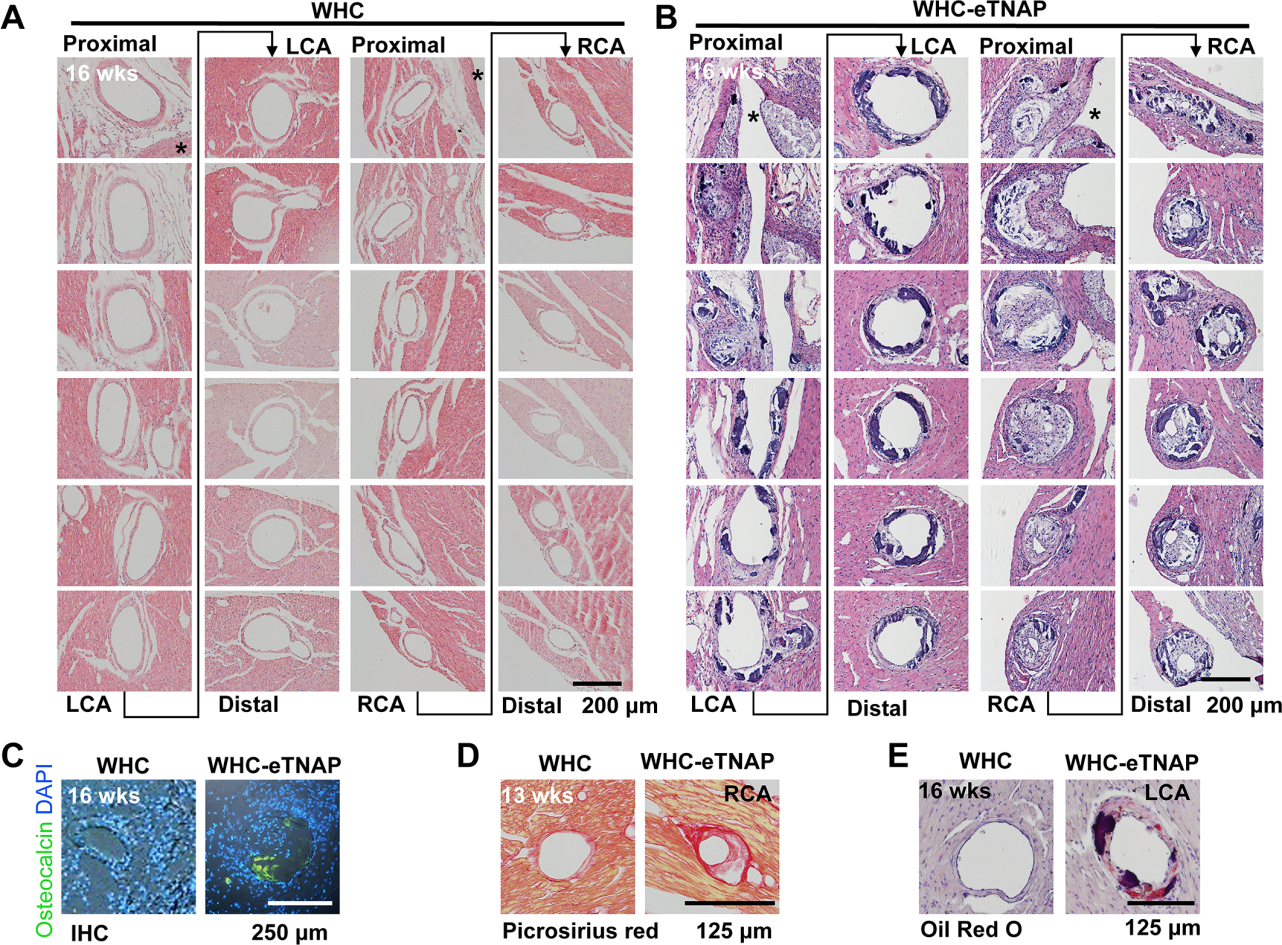
Coronary artery lesions in WHC-eTNAP mice. **(A)** Consecutive sections of a left coronary artery (LCA) and a septal branch of the right coronary artery (RCA) from a 16-weeks-old WHC mouse with hypercholesterolemia (H&E staining). Both vessels are unaffected by atherosclerosis. **(B)** Consecutive sections of LCA and RCA from a 16-weeks-old WHC-eTNAP mouse with hypercholesterolemia (H&E staining). Both vessels are affected by atherosclerosis, with the RCA being severely stenotic. **(C)** Immunohistochemical detection of osteocalcin (green) in 16-weeks-old WHC and WHC-eTNAP mice with hypercholesterolemia. Sections were counterstain with DAPI (blue). **(D)** Sections containing the RCA from 13-weeks-old WHC and WHC-eTNAP mice with hypercholesterolemia were stained with picrosirius red for collagen. **(E)** Oil red O staining (red) of the LCA from 16-weeks-old WHC and WHC-eTNAP mice with hypercholesterolemia, counterstained with hematoxylin (purple).

Histological examination of left and right coronary arteries from WHC and WHC-eTNAP mice was performed in serial sections stained with H&E. Left and right coronary arteries were traced in these sections spanning the heart base starting at coronary ostia (proximal) and extending over approximately 2–3 mm into the left ventricle free wall (main LCA) or the septum (RCA branch). This was performed in 16-weeks-old WHC and WHC-eTNAP mice with atherosclerosis. Coronary arteries from WHC mice were histologically normal (**Fig 4A**). LCA from WHC-eTNAP mice displayed intimal thickening, calcifications, and appeared dilated (**Fig 4B,** left panels). The septal branches, which in most animals in this study were originating from RCA ostia, were more severely affected (**Fig 4B** right panels). In the example shown, this septal branch of RCA was nearly completely occluded in proximal segments and severely stenotic in distal segments. Atherosclerotic plaques in proximal segments of RCA were less calcified compared to distal segments but contained more foam cells and necrotic core regions. More distal segments of RCA displayed a complex calcification pattern that appeared to be both medial and intimal. Macrophage foam cells were also detected in these segments. Immunohistochemistry revealed presence of the bone-specific marker osteocalcin within coronary lesions of 16 weeks old WHC-eTNAP mice on an athero diet (**Fig 4C**). Osteocalcin staining was absent in arteries from WHC mice on the same diet. Collagen staining with picrosirious red highlighted thin fibrous caps and unstained core regions in RCA lesions of a 13-weeks-old WHC-eTNAP mice on an athero diet; no lesions were detected in WHC mice on the same diet. Oil red O staining (with hemotoxylin counterstain) confirmed lipid deposition (red) within intimal thickening of LCA of 16-weeks-old WHC-eTNAP mice on an athero diet (**Fig 4E**); More lipids accumulated in opposition to calcifications (dark purple), which appeared as intimal nodules and medial sheets.

### Effect of TNAP inhibitor SBI-425 on the outcomes in WHC-eTNAP mice in the setting of hypercholesterolemia

We next sought to evaluate a potential therapeutic approach for the reduction of vascular calcification and atherosclerosis in this model, through the use of a specific TNAP inhibitor SBI-425 (30 mg*kg^-1^*d^-1^ in food). Three groups were studied: WHC + placebo, WHC-eTNAP + placebo, and WHC-eTNAP + SBI-425. All group were exposed to an atherogenic Paigen’s diet starting at 8 weeks of age and followed until 13 weeks of age.

#### Blood chemistry

**Fig 5:**
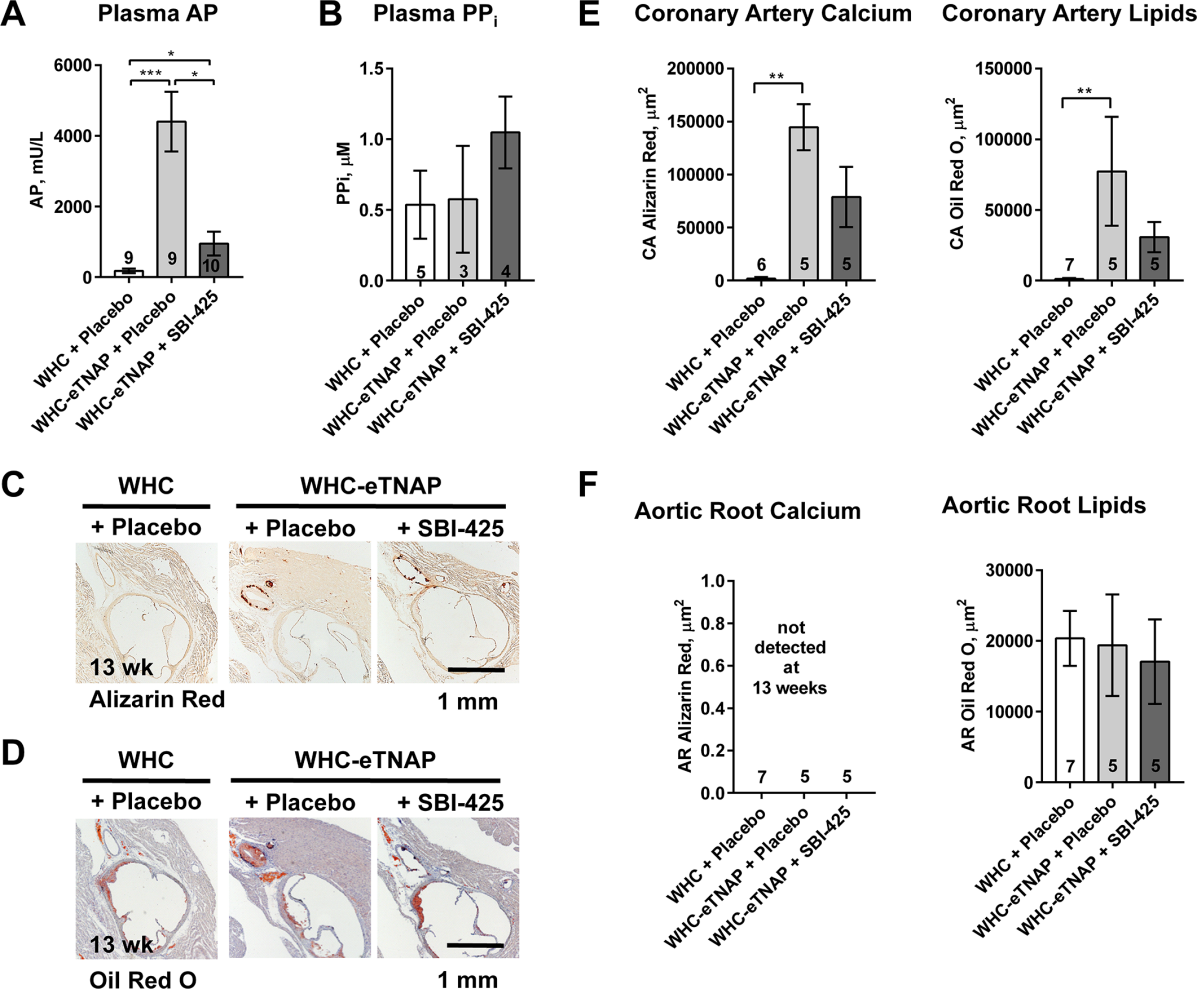
Effects of SBI-425 on plasma alkaline phosphatase activity, plasma pyrophosphate (PP_i_), and atherosclerosis in WHC-eTNAP mice. **(A)** Plasma alkaline phosphatase activity was measured in plasma from non-fasted mice collected one to three weeks after initiation of the treatment protocol. **(B)** Plasma PP_i_ was measured in plasma from non-fasted mice collected from a subset of animals one week after initiation of the treatment protocol. **(C)** Alizarin red staining for calcium is shown, representative images. **(D)** Oil red O staining, hematoxylin counterstained; representative images. **(E)** Quantification of calcium (based on alizarin red staining) and lipids (Oil red O staining) in coronary arteries. **(F)** Quantification of calcium (alizarin red staining) and lipids (Oil red O staining) in aortic roots. (C-F) Data were collected at 13 weeks of age; *, p < 0.05; **, p < 0.01; ***, p < 0.001.

Alkaline phosphatase levels were measured in plasma from non-fasted mice within one to three weeks after initiation of the treatment protocol. WHC-eTNAP + SBI-425 group achieved a significant reduction of plasma alkaline phosphatase activity compared to WHC-eTNAP + placebo (952 vs. 4404 mU/L, p<0.05). However, it remained significantly higher than in the WHC + placebo group, which served in this study as a coronary disease-free “healthy” control (952 vs. 178 mU/L, p < 0.05; **Fig 5A**). Pyrophosphate (PP_i_) levels were measured in plasma of a separate group of animals one week after initiation of treatments. PP_i_ was higher in SBI-425 treated mice compared to other groups (WHC-eTNAP + SBI-425: 1.05 μM; WHC-eTNAP + placebo: 0.57 μM; WHC + placebo: 0.54 μM). This increase, however, has not reached statistical significance assessed by ANOVA, followed my multiple comparisons (**Fig 5B**). There were no observed differences in plasma calcium, inorganic phosphate (P_i_), TG, or total cholesterol between groups (**Table 4**).

**Table 4:** Effect of SBI-425 on plasma calcium, phosphorus, and lipids (Mean ± SD).

Parameter	WHC + Placebo	WHC-eTNAP + Placebo	WHC-eTNAP + SBI-425
N	9	5	7
Ca, mg/dl	11.6 ± 2.0	11.2 ± 0.9	11.2 ± 1.7
P_i_, mg/dl	11.1 ± 4.7	7.5 ± 2.2	8.8 ± 2.9
TG, mg/dl	257 ± 156	161 ± 60	199 ± 131
CHOL, mg/dl	1466 ± 963	1205 ± 442	1411 ± 450

Ca, calcium; P_i_, phosphorus; TG, triglycerides; CHOL, cholesterol

#### Physiology and histology

Physiologic parameters were measured at baseline (8 weeks of age) and at 13 weeks of age. There was no difference between groups at baseline. Decrease survival was noted in WHC-eTNAP + placebo group compared to WHC + placebo (56% vs. 100% in WHC + placebo, p<0.05, **Table 5**). At 13 weeks of age, the WHC-eTNAP + placebo group exhibited a significant reduction in body weight compared to WHC + placebo mice (18.8 vs. 25.7 g, p<0.001). Their cardiac ejection fraction was also reduced (47% vs. 68%, p<0.05), whereas left ventricular mass to body weight ratio was increased compared to WHC + placebo (4.3 vs. 3.0, p<0.05). Coronary calcium and lipids were measured in alizarin red (**Fig 5C**) and oil red O-stained histological sections (**Fig 5D**). Coronary calcium and lipids were increased in the WHC-eTNAP + placebo group compared to WHC + placebo mice (calcium: 144622 vs. 1809 μm^2^, p<0.01; lipids: 77317 vs. 1173 μm^2^, p<0.01; **Fig 5E**).

**Table 5:** Physiologic parameters of WHC and WHC-eTNAP mice in the SBI-425 study (Mean ± SD).

Parameter	Time-point	WHC + Placebo	WHC-eTNAP + Placebo	WHC-eTNAP + SBI-425
N	Baseline	9	9	10
	13 wk	9	5	8
Survival, %	13 wk	100	56*	80
BW, g	Baseline	24.9 ± 1.2	24.0 ± 1.5	24.0 ± 2.2
	13 wk	25.7 ± 3.4	18.8 ± 2.7***	22.4 ± 4.2
HR, bpm	Baseline	391 ± 52	423 ± 62	413 ± 60
	13 wk	441 ± 65	395 ± 72	452 ± 92
LV EDD, mm	Baseline	3.7 ± 0.1	3.8 ± 0.4	3.7 ± 0.4
	13 wk	3.7 ± 0.3	3.8 ± 0.7	3.4 ± 0.6
EF, %	Baseline	66 ± 5	69 ± 9	67 ± 10
	13 wk	68 ± 10	47 ± 16*	59 ± 17
CO/BW, ml*min^-1^*g^-1^	Baseline	0.52 ± 0.13	0.66 ± 0.22	0.57 ± 0.13
	13 wk	0.59 ± 0.12	0.58 ± 0.31	0.48 ± 0.19
LV mass/BW, mg*g^-1^	Baseline	3.7 ± 0.8	3.5 ± 0.6	3.5 ± 0.8
	13 wk	3.0 ± 0.6	4.3 ± 1.4*	3.8 ± 1.2

BW, body weight; HR, heart rate; LV ESD, end systolic diameter of the left ventricle; LV EDD, end diastolic diameter of the left ventricle; EF, ejection fraction; CO, cardiac output; LV mass, left ventricular mass

* p < 0.05

*** p < 0.001 vs. WHC + Placebo

SBI-425 treatment partially normalized all these parameters. In the WHC-eTNAP + SBI 425 group survival was increased to 80%; body weight was 22.4 g; ejection fraction was 59%; and left ventricular mass to body weight ratio was 3.8 (**Table 5**). Furthermore, inhibition of TNAP by SBI-425 resulted in a 1.8-fold reduction in coronary calcium and a 2.5-fold reduction in coronary lipids (calcium: 78838 μm^2^; lipids: 30754 μm^2^ **Fig 5E**). Although all these interesting effects were significant in a direct pair-wise comparison between WHC-eTNAP + placebo and WHC-eTNAP + SBI-425 groups, the effects disappeared when the WHC + placebo group was included in the model (as it was pre-specified by the study design).

Aortic root calcium was not detected in this experiment. Lipid-positive areas in aortic root plaques were similar between all groups (WHC + placebo: 20363 μm^2^, WHC-eTNAP + placebo: 19349 μm^2^; WHC-eTNAP + SBI-425: 17069 μm^2^; **Fig 5F**).

## Discussion

The results of this study support our hypothesis that “primary” calcified lesions in the intima can serve as permissible sites for lipid deposition and can initiate the development of complex plaques. Whether such a hypothetical mechanism operates in human vasculature and under what conditions endothelial TNAP activity might become elevated remains unknown. In a mouse model, this unusual course of coronary atherosclerosis requires significant elevation of plasma cholesterol to the level rarely occurring in human patients [38].

To create hypecholesterolemic conditions we chose to use a mouse model harboring a point mutation in the LDL receptor (LDLR), named *wicked high cholesterol*, *WHC* [22]. *WHC* affects a conserved cysteine residue reported in multiple human genetic studies of familial hypercholesterolemia [39–41]. The murine WHC mutation was discovered at The Jackson Laboratory though a chemical mutagenesis screen aimed to identify new recessive mutations associated with significant cardiovascular and metabolic phenotypes [42]. A complementation cross between WHC and complete LDLR-deficient was performed as part of the original study and resulted in F1 progeny with plasma cholesterol levels essentially identical to those in homozygous *ldlr* knockouts therefore confirming that WHC and *ldlr* knockout alleles are functionally equivalent [22].

Endogenous TNAP can be localized with endothelial in tissues based on the detection of *in situ* alkaline phosphatase (AP) activity. AP activity is expressed in terminal pre-capillary arterial tree in multiple tissues [29] and in endothelium of larger arteries in skeletal muscle of many laboratory animals and humans [30]. Very little is known about the distribution and function of TNAP in human coronary circulation in health and disease states [31]. In this study we documented that AP activity is expressed in small arteries and in microvascular endothelial cells in human myocardium and, at least in one case, it co-localized with arterial calcification. AP activity in human myocardium was sensitive to inhibition by 12.5 mM L-homoarginine, a specific inhibitor that was used in older biochemical studies to distinguish TNAP from other alkaline phosphatases [28]. The presence of endothelial TNAP in human tissues in conjunction with our observation of the role that TNAP might play in the pathophysiology of vascular calcification, and availability of a specific inhibitor of TNAP with pharmacological properties (SBI-425), may warrant a further investigation into pathophysiological mechanisms of vascular disease that involve endothelial TNAP function.

Studies in mice examining the effects of calcification on atherosclerotic suggest that intimal calcification may not have an effect on plaque expansion. For example, the VSMC-specific deficiency of *Runx2*, a positive regulator of osteoblast differentiation [43, 44], reduces calcification of atherosclerotic plaques in dyslipidemic *apoE* knockout mice, but fails to halt progression of aortic atherosclerosis in this model [45]. OPG, a protein that inhibits vascular calcification [46, 47], was shown to reduce atherosclerosis in apoE knockout mice [48]. However in another model (*ldlr* knockout mice), OPG reduced calcification, but had no effect on the size of atherosclerotic plaques [49]. Our study suggests that TNAP activity might promote secondary calcification of *bona fide* atherosclerotic plaque in the aortic root, but this excess of calcification does not result in larger lesions. However, we also observed that pre-existing intimal calcification in coronary arteries appears to promote plaque formation. The latter mechanism deviates from normal temporal progression of atherosclerosis [32].

eTNAP mice exhibited massive upregulation of circulating and tissue TNAP activity, which should, theoretically, result in the reduction of plasma PP_i_. However, reduction of plasma PP_i_ was not observed in this study nor in the previous reports of TNAP overexpression in osteoblasts, vascular smooth muscle cells, or endothelial cells [10, 11, 50]. We have speculated that the reduction in circulating PP_i_ levels could be partially compensated by upregulation of PP_i_ production, thus sustaining elevated TNAP activity [11]. This suggestion was based on the fact that expression of the PP_i_‐producing enzyme ectonucleotide pyrophosphatase/phosphodiesterase 1 (Enpp1) was upregulated in TNAP transgenics compared with wild type mice [10]. Another question is whether the local or systemic TNAP activity drives vascular calcification. Epidemiological studies clearly show that increased blood alkaline phosphatase activity correlates with coronary artery calcification [15, 16]. Therefore, it is plausible that circulating enzyme can be deposited into vascular tissues to increase calcification. We report here that TNAP overexpression and concomitant increase in plasma AP activity increases secondary intra-plaque calcification in aortic root plaques in 16-weeks-old mice. Further studies are needed to ascertain that any source of plasma TNAP can increase plaque calcification.

Our study has several limitations, (1) supra-physiological expression of TNAP and incomplete inhibition of AP activity by SBI-425. It would be interesting to test this inhibitor in other models of vascular calcification not involving direct overexpression of TNAP. This idea is interesting because TNAP plays a central role in bio-mineralization and therefore can be viewed as a mechanistic (and therapeutic) node in vascular calcification. (2) Potential side-effects of TNAP inhibition on the skeletal system. Oral treatment of adult animals with SBI-425 was well-tolerated over the course of five weeks but effects of SBI-425 on bone health were not tested in this study. In our original study of SBI-425, daily injections, administered starting from birth, had no detectable skeletal side effects in young growing animals [10]. In general, the challenges of using TNAP inhibition for treatment of vascular calcification would undoubtedly involve the protection of skeletal system from side effects such as osteoporosis. This might require targeted delivery and/or vascular specific activation of pro-drugs.

In conclusion, we established that atherogenesis can take an unusual course in which primary intimal calcification in a form of sub endothelial nodules can interact with hypercholesterolemia and increase lipid deposition into such lesions. The relevance of this mechanism observed in an animal model to human vascular disease is not clear.

## Supporting information

S1 Fig A wicked high cholesterol model (WHC) model of atherosclerosis. **(A)** WHC and C57BL/6J mice (WT) were treated with an atherogenic Paigen’s starting at 8 weeks of age; plasma cholesterol (CHOL) was measured at 13 and 23 weeks of age. **(B)** Plasma lipoprotein profiles were determined by size exclusion chromatography at 23 weeks of age; plasma was pooled from 3 animals per group. **(C)** Quantification of aortic root atherosclerosis (expressed as % aortic root occlusion) and calcification (expressed as the area of positive alizarin red staining); experiments in A-C were conducted in both sexes; data for male mice are shown. **(D)** Representative images of the aortic roots of WHC male mice at baseline (8 weeks of age) and at 23 weeks of age (15 weeks on an Paigen’s diet); serial sections were stained for lipids (oil red O with hematoxylin counterstain); AP activity (BCIP/NBT alkaline phosphatase substrate kit); and calcium (alizarin red). **(E)** Representative images of coronary arteries from the same aortic rood samples as in D.
(TIF)

S2 Fig Alkaline phosphatase staining in a panel of tissues from WHC and WHC-eTNAP mice on an atherogenic diet at 16 weeks of age. **(A)** Right coronary artery ostium; **(B)** aortic root; arrows demarcate plaque. **(C)** Aorta. **(D)** Mesentery. **(E)** Lung.
(TIF)

S3 Fig Cytokines in plasma. **(A)** TNFα. **(B)** IL-1β. There were no differences detected between 13 and 16-weeks-old mice; data were pooled from two age groups; *, p < 0.05.
(TIF)

S4 Fig Atherosclerosis in coronary arteries and aortic roots of WHC and WHC-eTNAP mice on a modified Paigen’s diet without cholate. **(A)** Alizarin red staining for calcium, representative images. **(B)** Oil red O staining, hematoxylin counterstained; representative images. **(C)** Quantification of calcium (based on the alizarin red staining) and lipids (Oil red O staining). **(D)** Quantification of calcium (alizarin red staining) and lipids (Oil red O staining) in the aortic root. All data were collected at 13 weeks of age.
(TIF)

S5 Fig Runx2 qPCR and immunohistochemistry. **(A)** Runx2 qPCR results. **(B)** Runx2 immunohistochemistry. Representative images from aortic roots and coronary arteries of WHC and WHC-eTNAP mice at baseline (8 weeks of age). Tissues were stained with a rat monoclonal anti-Runx2 antibody or a secondary antibody only (anti-rat IgG) followed by immunoperoxidase detection and hematoxylin counterstaining.
(TIF)

S1 Table Physiologic characteristics of wild type (B6) and WHC mice at baseline and on an atherogenic Paigen’s diet at 13 and 23 weeks of age (Mean ± SD). (DOCX)

S2 Table Physiologic characteristics of WHC and WHC-eTNAP mice at baseline and on an atherogenic modified Paigen’s diet (without cholate) at 13 weeks of age (Mean ± SD). (DOCX)

S1 File Supplemental methods. (DOCX)
