# Supplementary figures and images for: Overexpression of tissue-nonspecific alkaline phosphatase (TNAP) in endothelial cells accelerates coronary artery disease in a mouse model of familial hypercholesterolemia
Source: PLoS One. 2017 Oct 12;12(10):e0186426. doi: 10.1371/journal.pone.0186426 (PMC5638543; doi:10.1371/journal.pone.0186426)

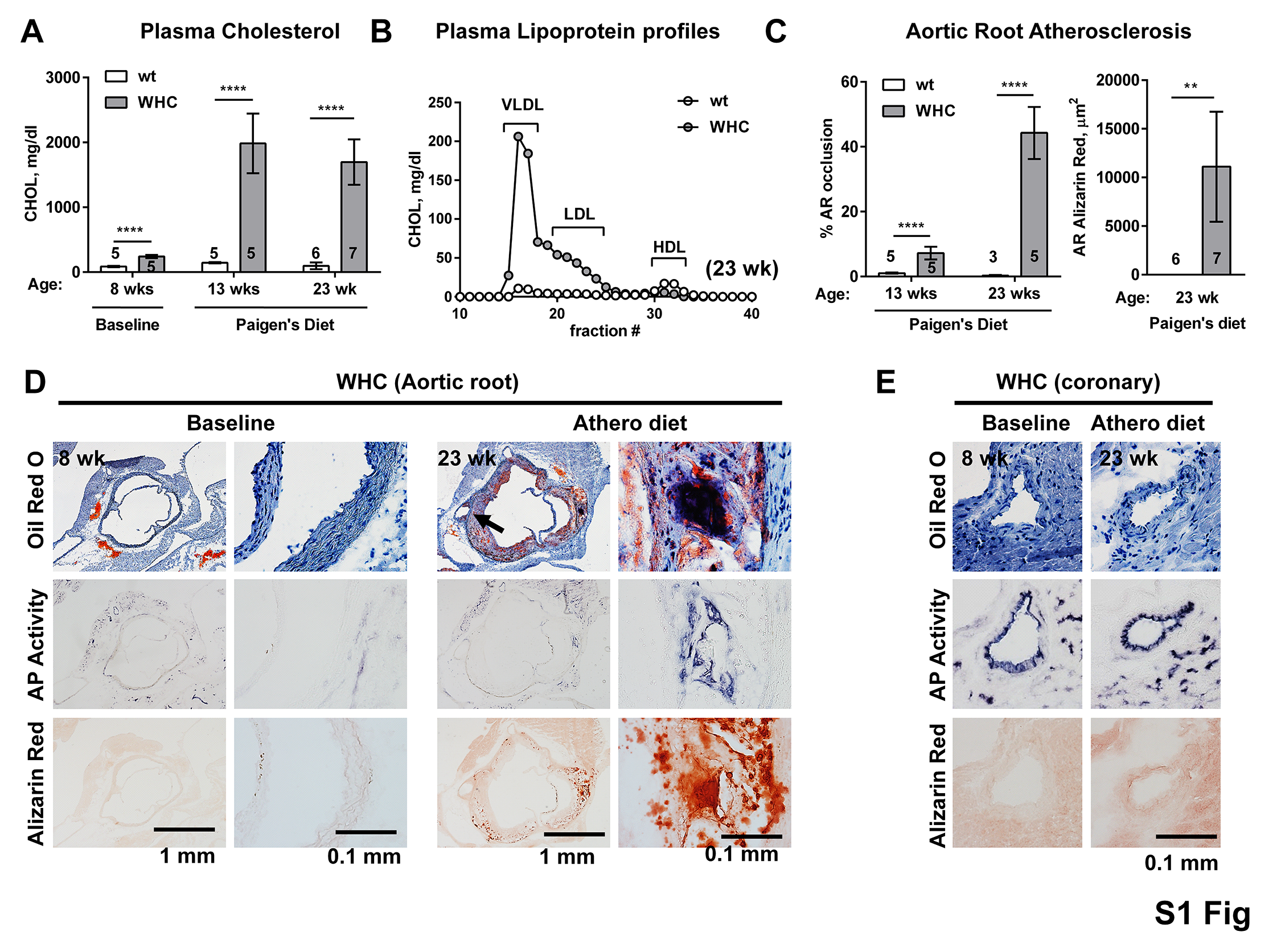

Supplement: S1 Fig — (A) WHC and C57BL/6J mice (WT) were treated with an atherogenic Paigen’s starting at 8 weeks of age; plasma cholesterol (CHOL) was measured at 13 and 23 weeks of age. (B) Plasma lipoprotein profiles were determined by size exclusion chromatography at 23 weeks of age; plasma was pooled from 3 animals per group. (C) Quantification of aortic root atherosclerosis (expressed as % aortic root occlusion) and calcification (expressed as the area of positive alizarin red staining); experiments in A-C were conducted in both sexes; data for male mice are shown. (D) Representative images of the aortic roots of WHC male mice at baseline (8 weeks of age) and at 23 weeks of age (15 weeks on an Paigen’s diet); serial sections were stained for lipids (oil red O with hematoxylin counterstain); AP activity (BCIP/NBT alkaline phosphatase substrate kit); and calcium (alizarin red). (E) Representative images of coronary arteries from the same aortic rood samples as in D. (TIF) [file pone.0186426.s001.tif]

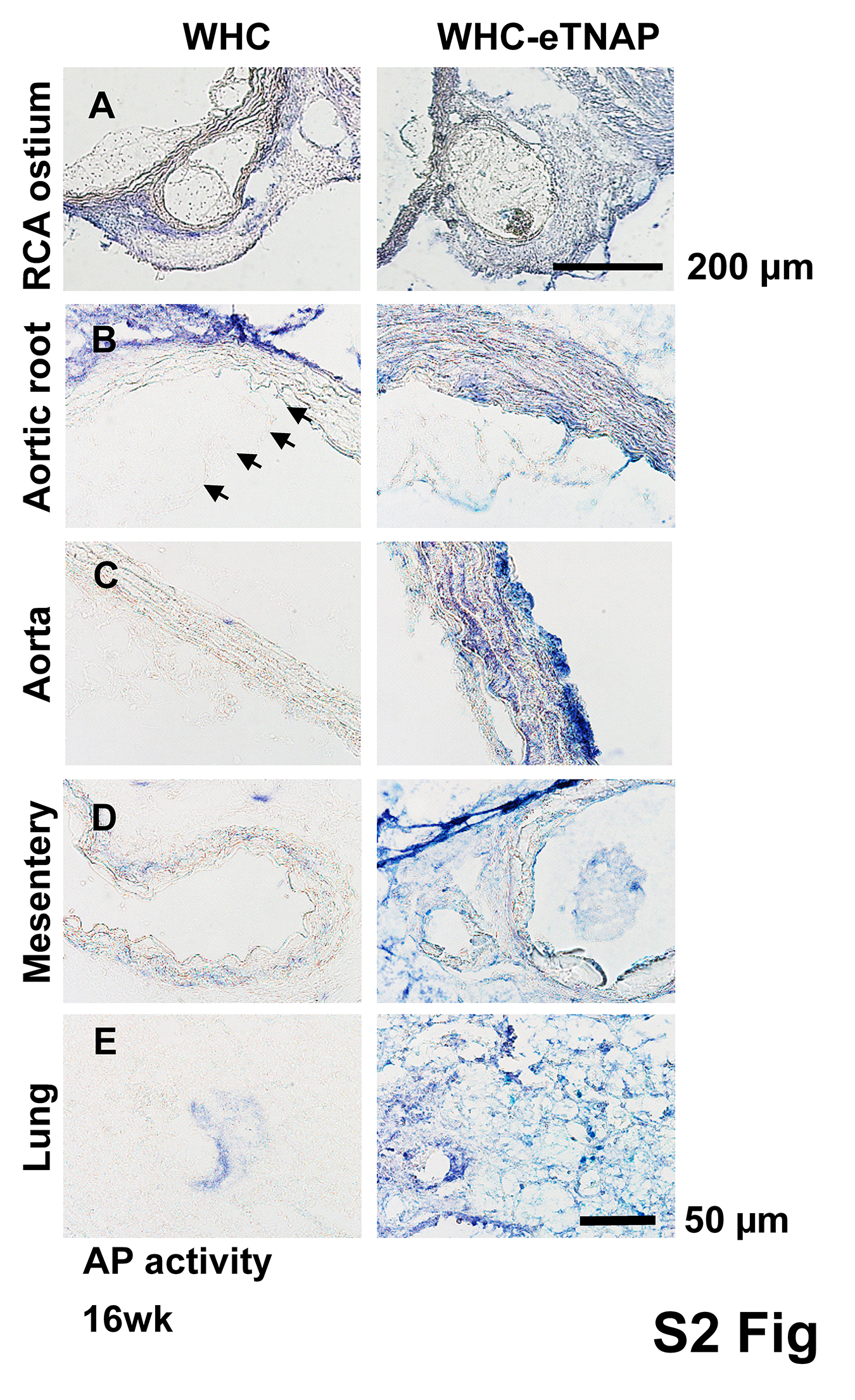

Supplement: S2 Fig — (A) Right coronary artery ostium; (B) aortic root; arrows demarcate plaque. (C) Aorta. (D) Mesentery. (E) Lung. (TIF) [file pone.0186426.s002.tif]

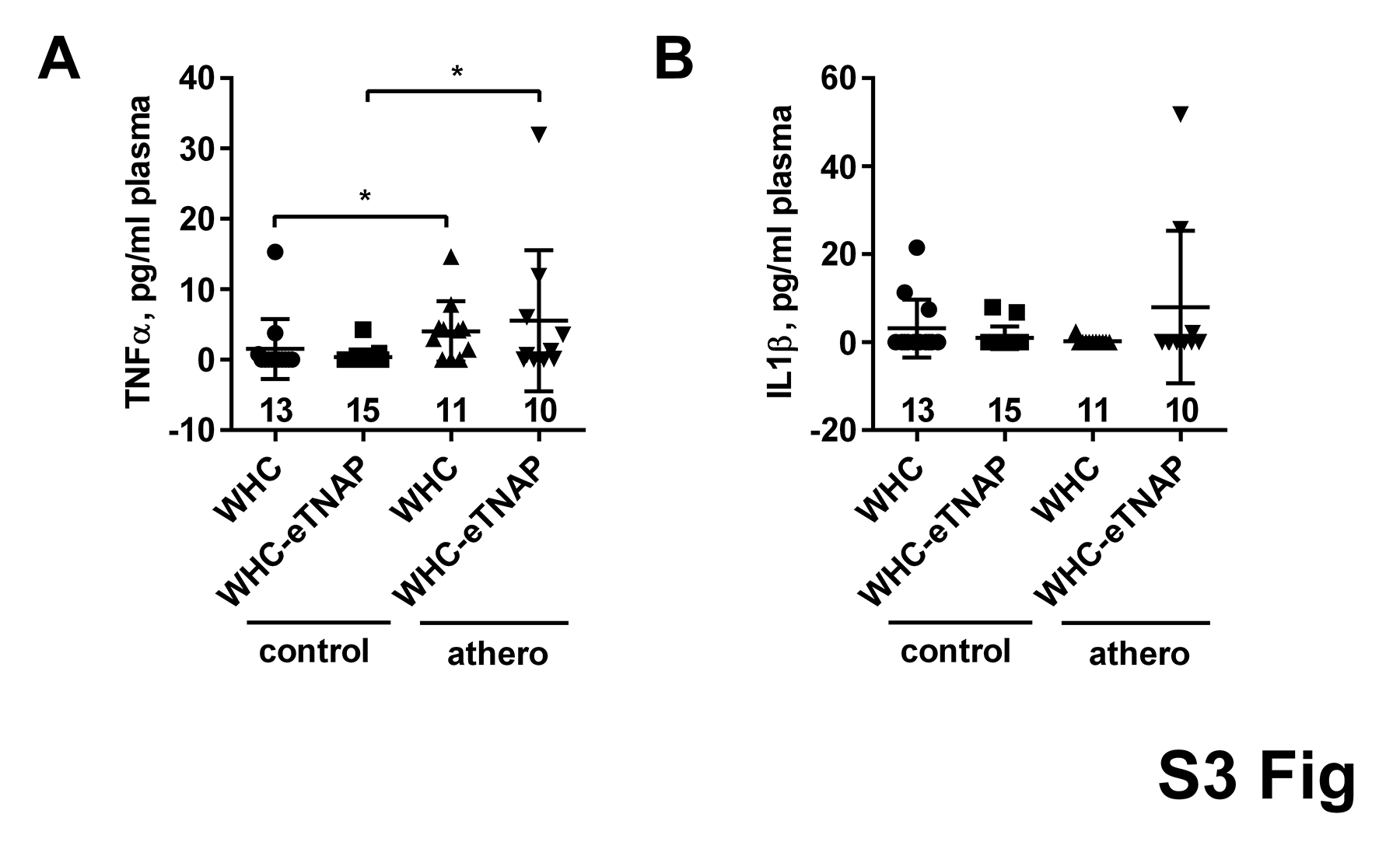

Supplement: S3 Fig — (A) TNFα. (B) IL-1β. There were no differences detected between 13 and 16-weeks-old mice; data were pooled from two age groups; *, p < 0.05. (TIF) [file pone.0186426.s003.tif]

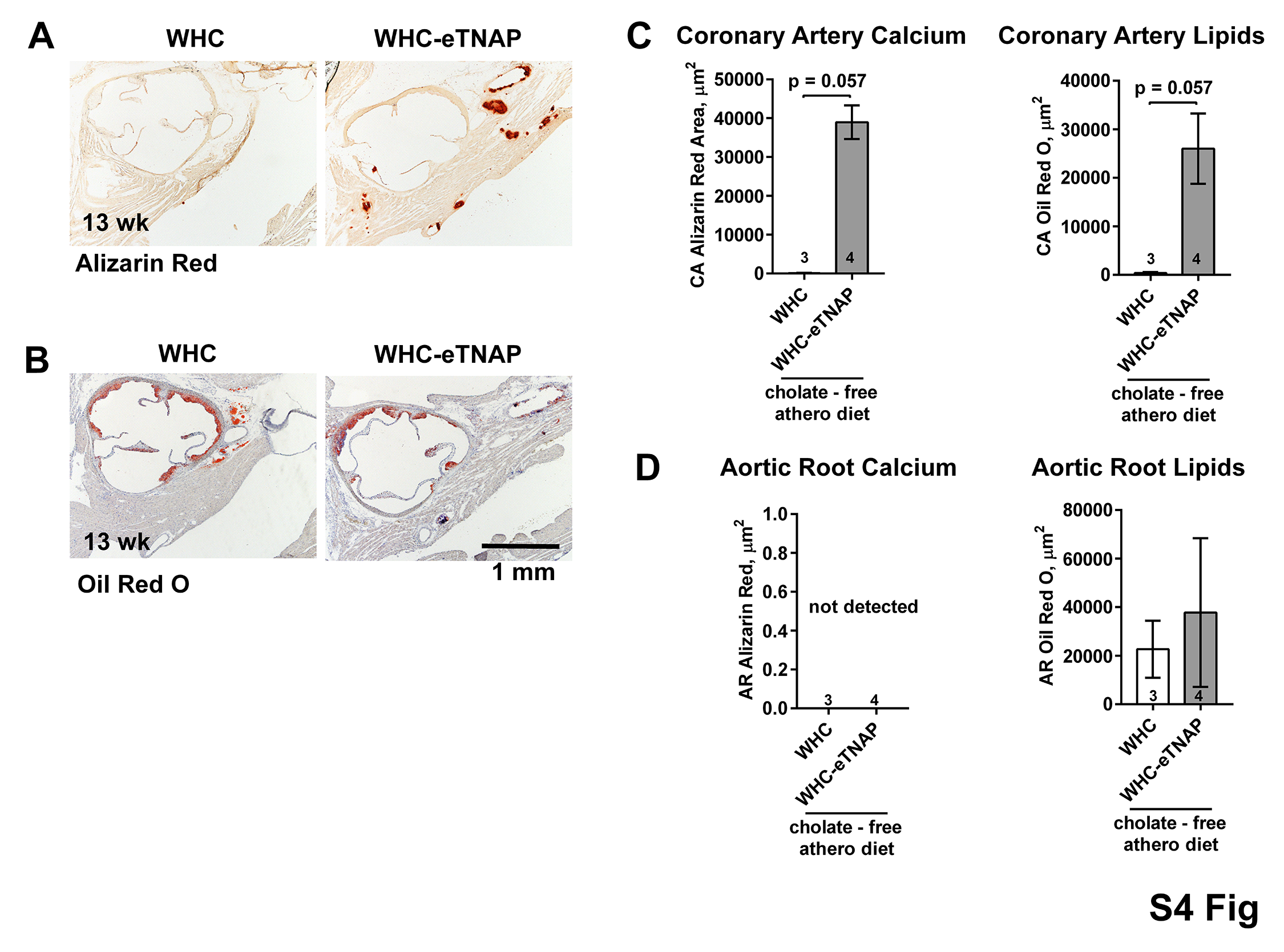

Supplement: S4 Fig — (A) Alizarin red staining for calcium, representative images. (B) Oil red O staining, hematoxylin counterstained; representative images. (C) Quantification of calcium (based on the alizarin red staining) and lipids (Oil red O staining). (D) Quantification of calcium (alizarin red staining) and lipids (Oil red O staining) in the aortic root. All data were collected at 13 weeks of age. (TIF) [file pone.0186426.s004.tif]

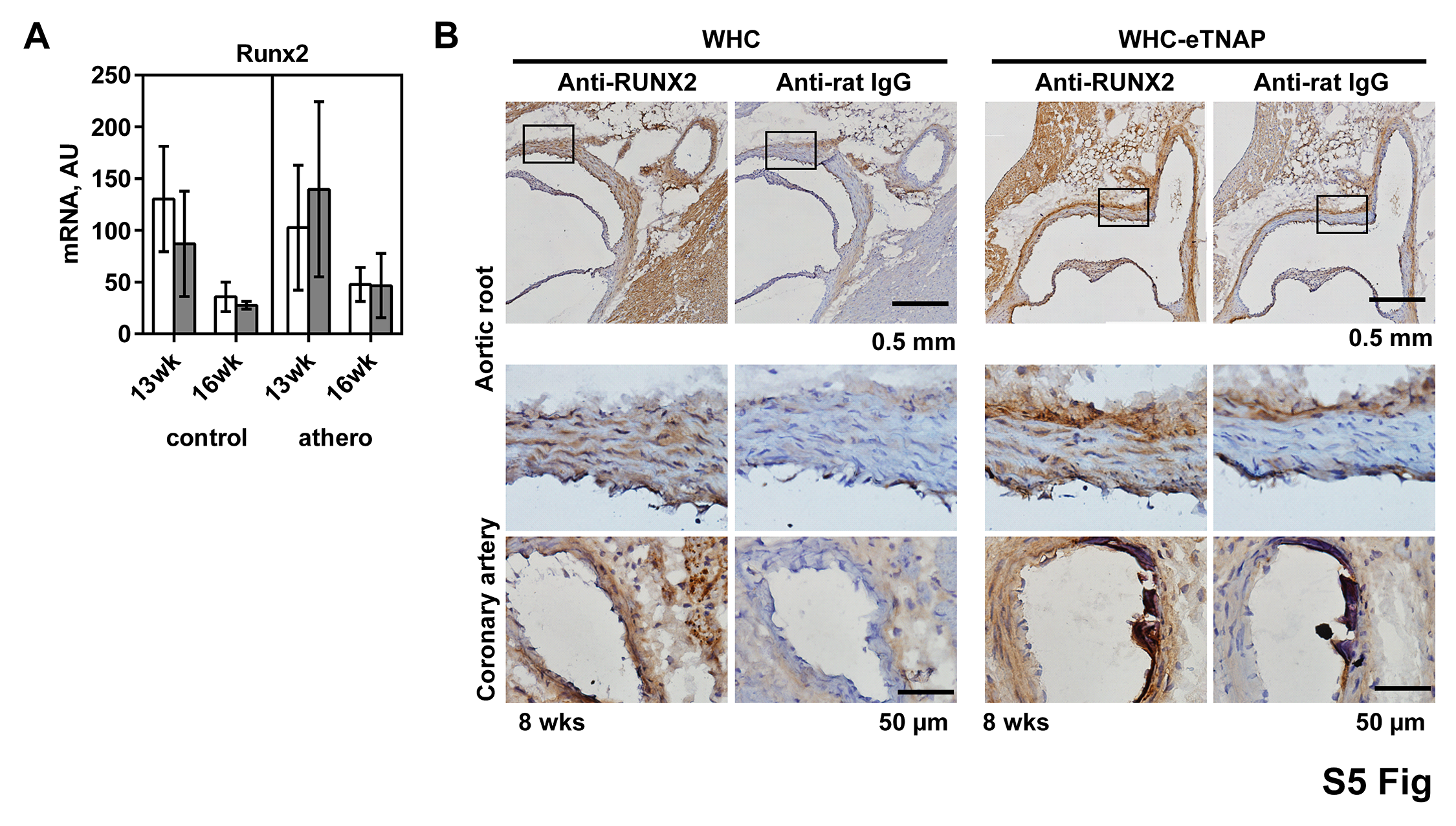

Supplement: S5 Fig — (A) Runx2 qPCR results. (B) Runx2 immunohistochemistry. Representative images from aortic roots and coronary arteries of WHC and WHC-eTNAP mice at baseline (8 weeks of age). Tissues were stained with a rat monoclonal anti-Runx2 antibody or a secondary antibody only (anti-rat IgG) followed by immunoperoxidase detection and hematoxylin counterstaining. (TIF) [file pone.0186426.s005.tif]
