# Supplementary material for: Overexpression of tissue-nonspecific alkaline phosphatase (TNAP) in endothelial cells accelerates coronary artery disease in a mouse model of familial hypercholesterolemia
Source: PLoS One. 2017 Oct 12;12(10):e0186426. doi: 10.1371/journal.pone.0186426 (PMC5638543; doi:10.1371/journal.pone.0186426)
Supplement: S1 Table — (DOCX) [file pone.0186426.s006.docx]

**S1 Table.** Physiologic characteristics of wild type (B6) and WHC mice at baseline and on an atherogenic Paigen’s diet at 13 and 23 weeks of age (Mean ± SD)

| Parameter | Age group | wt | WHC |
| --- | --- | --- | --- |
| N | baseline | 9 | 9 |
|  | 13 wk | 9 | 9 |
|  | 23 wk | 6 | 7 |
| BW, g | baseline | 25.1 ± 0.8 | 23.5 ± 2.1 |
|  | 13 wk | 26.8 ± 0.7 | 24.0 ± 1.6*** |
|  | 23 wk | 28.3 ± 1.1 | 25.6 ± 1.7** |
| HR, bpm | baseline | 444 ± 43 | 441 ± 35 |
|  | 13 wk | 448 ± 28 | 420 ± 21 |
|  | 23 wk | 398 ± 35 | 417 ± 28 |
| LV EDD, mm | baseline | 3.7 ± 0.3 | 3.5 ± 0.2 |
|  | 13 wk | 3.9 ± 0.2 | 3.6 ± 0.2* |
|  | 23 wk | 4.2 ± 0.3 | 4.0 ± 0.2 |
| EF, % | baseline | 70 ± 6 | 66 ± 8 |
|  | 13 wk | 58 ± 7 | 60 ± 6 |
|  | 23 wk | 52 ± 11 | 54 ± 5 |
| CO/BW, ml*min^-1^*g^-1^ | baseline | 0.69 ± 0.12 | 0.63 ± 0.08 |
|  | 13 wk | 0.64 ± 0.10 | 0.58 ± 0.06 |
|  | 23 wk | 0.57 ± 0.08 | 0.59 ± 0.04 |
| LVmass/BW, mg*g^-1^ | baseline | 3.4 ± 0.5 | 3.1 ± 0.2 |
|  | 13 wk | 3.0 ± 0.1 | 2.9 ± 0.2 |
|  | 23 wk | 3.0 ± 0.2 | 2.8 ± 0.2 |

BW, body weight; HR, heart rate; LV EDD, end diastolic diameter of the left ventricle; EF, ejection fraction; CO, cardiac output; LV mass, left ventricular mass; *, p < 0.05, **, p < 0.01, ***, p < 0.001 vs. the same age wild type mice. Experiments were conducted in both sexes; data for male mice are shown.
