# Supplementary material for: Overexpression of tissue-nonspecific alkaline phosphatase (TNAP) in endothelial cells accelerates coronary artery disease in a mouse model of familial hypercholesterolemia
Source: PLoS One. 2017 Oct 12;12(10):e0186426. doi: 10.1371/journal.pone.0186426 (PMC5638543; doi:10.1371/journal.pone.0186426)
Supplement: S2 Table — (DOCX) [file pone.0186426.s007.docx]

**S2 Table.** Physiologic characteristics of WHC and WHC-eTNAP mice at baseline and on a modified Paigen’s diet without cholate at 13 weeks of age (Mean ± SD)

| Parameter | Age group | WHC | WHC-eTNAP |
| --- | --- | --- | --- |
| N | baseline | 6 | 5 |
|  | 13 wk | 6 | 6 |
| BW, g | baseline | 24.7 ± 0.9 | 25.4 ± 2.4 |
|  | 13 wk | 29.7 ± 1.4 | 24.7 ± 3.6* |
| HR, bpm | baseline | 395 ± 38 | 398 ± 18 |
|  | 13 wk | 457 ± 45 | 389 ± 86 |
| LV EDD, mm | baseline | 3.7 ± 0.2 | 4.0 ± 0.2 |
|  | 13 wk | 3.8 ± 0.3 | 3.9 ± 0.5 |
| EF, % | baseline | 65 ± 10 | 61 ± 9 |
|  | 13 wk | 68 ± 6 | 60 ± 11 |
| CO/BW, ml*min^-1^*g^-1^ | baseline | 0.55 ± 0.08 | 0.63 ± 0.16 |
|  | 13 wk | 0.58 ± 0.16 | 0.60 ± 0.21 |
| LVmass/BW, mg*g^-1^ | baseline | 3.8 ± 0.3 | 4.2 ± 0.5 |
|  | 13 wk | 3.3 ± 0.7 | 3.9 ± 1.4 |

BW, body weight; HR, heart rate; LV EDD, end diastolic diameter of the left ventricle; EF, ejection fraction; CO, cardiac output; LV mass, left ventricular mass; *, p < 0.05 vs. the same age WHC
