# Supplementary material for: Overexpression of tissue-nonspecific alkaline phosphatase (TNAP) in endothelial cells accelerates coronary artery disease in a mouse model of familial hypercholesterolemia
Source: PLoS One. 2017 Oct 12;12(10):e0186426. doi: 10.1371/journal.pone.0186426 (PMC5638543; doi:10.1371/journal.pone.0186426)
Supplement: S1 File — (DOCX) [file pone.0186426.s008.docx]

**Supplemental methods**

**Size exclusion chromatography**

Plasma was pooled from three animals per group and filtered through 0.45 µm filter. 300 µl of filtered plasma was injected into Superose 6 10/300GL column (GE Healthcare) equilibrated with phosphate buffered saline (PBS). Column was developed in PBS at 0.25 ml/min flow rate; 0.5 ml fractions were collected, 20 µl from each fraction were used to measure cholesterol concentration using a plate reader.

**Immunohistochemistry**

Runx2 was visualized by immunohistochemistry in aortic root cryosections from 8-weeks old WHC and WHC-eTNAP mice using rat monoclonal antibody (item MAB2006; R&D Systems) followed by HRP–labeled goat anti‐rat IgG antibody (item sc-2006; Santa Cruz Biotechnology) and a retection with ImmPACT DAB Peroxidase substrate (Item SK-4105, Vector Labs). Sections were counterstained with hematoxylin.

**Cytokines measurements**

TNFα and IL-1β were measured in plasma using ELISA kits from eBiosciences according to manufacturer recommendations (TNFα - Item #88-7324-22, IL-1β - Item # 88-7013-22; ThermoFisher).
